# To Detach or Not to Detach? Two Experimental Studies on the Affective Consequences of Detaching From Work During Non-work Time

**DOI:** 10.3389/fpsyg.2020.560156

**Published:** 2020-10-16

**Authors:** Sabine Sonnentag, Cornelia Niessen

**Affiliations:** ^1^Department of Psychology, University of Mannheim, Mannheim, Germany; ^2^Chair of Work and Organizational Psychology, Institute of Psychology, Friedrich–Alexander University Erlangen–Nürnberg, Erlangen, Germany

**Keywords:** recovery, psychological detachment, negative affect, positive affect, experiment

## Abstract

Previous correlational studies have shown that both psychological detachment from work and positively thinking about work during non-work time are associated with favorable affective states. In our research we integrate these contradictory findings and add more rigor to detachment research by using an experimental design. In two experimental studies conducted in the laboratory, we manipulated two different kinds of detachment from work (thinking about a hobby; explicit detachment instruction) and three different kinds of thinking about work (thinking negatively, thinking positively, thinking in an unspecific way) by short written instructions. Results show that both detachment strategies lead to a reduction in negative affect (in both studies) and to an increase in positive affect (in one study). The effect of detachment was particularly strong when it was contrasted with thinking negatively about work and when end-of-workday negative affect was high. In some of the comparisons, the affective benefits of positively thinking about work were stronger than those of psychological detachment from work. Taken together, our studies demonstrate that detachment from work as well as positive thinking improves subsequent affect, highlighting the causality underlying the association between psychological detachment from work – as a core recovery experience – and subsequent affective states.

## Introduction

Today’s work situations are often highly demanding and ask for effective recovery processes during after-work hours. Research has identified psychological detachment from work during non-work time as an important feature of a successful recovery process, with psychological detachment being associated with favorable affective states in the short term and high well-being in the longer term (Sonnentag and Fritz, 2015). For instance, when employees do not think about work in the evening, but “switch off” and get a mental break they experience lower levels of negative and higher levels of positive states when they return to work in the next morning (Sonnentag et al., 2008; ten Brummelhuis and Bakker, 2012).

Overall, the recovery and detachment literature has drawn a rather positive picture of psychological detachment from work as an important recovery experience (Bennett et al., 2018). This positive perspective, however, neglects potential differences between various ways of thinking about work during after-work hours and tends to ignore conflicting findings from studies that demonstrated affective benefits of positively thinking about work during after-work hours (Meier et al., 2016; Clauss et al., 2018). With our present research we question the undifferentiated positive view on psychological detachment from work and point to potential downsides of fully detaching from work during after-work hours. Thus, we “push back” on the idea that mentally disconnecting from work is the best option for achieving favorable affective states during after-work hours.

Research has shown that affective states play an important role at the interface between work and non-work life, with affect at the end of work coloring affect experienced at home (Eby et al., 2010). Research on psychological detachment, however, has largely ignored affective states at the end of work when examining the benefits of psychological detachment. This oversight implies that rather little is known about when psychological detachment is highly needed and when is it of minor importance. To address this gap and learn more about the circumstances under which detachment is effective and beneficial for subsequent affect, we will examine if the affective state at the end of work is related to the benefit people gain from detaching from work. Specifically, we will test if end-of-work affect interacts with detachment in predicting change in subsequent affect.

Although previous research on psychological detachment from work is informative and highlights the importance of recovery during off-job time, studies so far have relied on correlational designs (Wendsche and Lohmann-Haislah, 2017; Bennett et al., 2018). Despite some studies used more sophisticated correlational designs relying on daily diary data (Sonnentag et al., 2008; Germeys and De Gieter, 2017) and panel studies (Sianoja et al., 2018), conclusions about causality remain ambiguous and research does not provide an unequivocal answer if psychological detachment indeed *causes* favorable affective states. For instance, when using correlational designs it is difficult to dismiss alternative explanations referring to third variables. To rule out alternative explanations and to establish causality between psychological detachment from work and change in subsequent affective states, an experimental approach is needed. Although a handful of intervention studies (e.g., Hahn et al., 2011; Ebert et al., 2015) overcame some of the limitations of correlational studies, conclusions to be drawn about psychological detachment remain limited because these intervention studies combined the psychological-detachment instruction with various other positive treatments, obscuring the unique effects psychological detachment might have.

To provide a more differentiated picture on the benefits and potential downsides of psychological detachment and to address questions of causality, we conduct two experimental studies in which we manipulate psychological detachment from work and compare the affective benefits of psychological detachment with the affective consequences of three different ways of thinking about the past day at work: (a) thinking about negative experiences, (b) thinking about positive experiences, and (c) thinking generally about the past day at work (i.e., “unspecific thinking”). With our studies, we address three research questions: First, we examine if detaching from work after the workday leads to a decrease in state negative and an increase in state positive affect, compared to thinking about work. Second, we test if the affective benefits of psychological detachment from work might be smaller than thinking positively about one’s work. Third, we examine if a person’s naturally occurring momentary affective state at the end of the workday matters for the affective benefits of detaching from work. Examining the effect of psychological detachment on state affect is important for several reasons. First, affect spills over across life domains (Eby et al., 2010) and is an important predictor of relevant experiences and behaviors in various domains (Rothbard and Wilk, 2011; Kempen et al., 2019). Second, changes in affect caused by (lack of) detachment may explain why detachment is important for longer-term health and well-being outcomes.

Our research offers important contributions to the literature. First, we add to research on recovery processes in general and psychological detachment in particular. We shed more light on the factors that drive the beneficial effects of psychological detachment and identify factors that explain when it might be better to continue thinking about work. Our research helps to gain insight into the specific features of not detaching from work that trigger the unfavorable outcomes usually associated with not detaching from work. Is it the fact that work-related content it still cognitively present during non-work time? Or is it a specific affective valence that accounts for the detrimental effect of not detaching? Moreover, by examining the role of state affect at the end of the workday as a potential moderator of the detachment effect, our research points to situations in which psychological detachment is particularly important.

Second, our research adds more rigor to the recovery literature by explicitly addressing the assumed causal effect of psychological detachment on subsequent affect. Addressing causality in an experimental design is important for theory building around job stress and employee recovery. Knowing if psychological detachment indeed *causes* subsequent affect will be important in developing a better understanding of the processes that help in undoing the negative effect of job stress on employee well-being.

Finally, our research will have implications for practice by offering information about how people should craft their leisure time (de Bloom et al., 2020). More specifically, our studies will provide guidance for employees if they should fully detach from work (e.g., for immersing themselves in a hobby or activities with family and friends) or if they may stay mentally connected to work by thinking about positive on-the-job experiences.

### The Detachment Concept

Etzion et al. (1998) characterized “sense of detachment” from work as “the individual’s sense of being away from the work situation” (p. 579). Psychological detachment is an experience that occurs during non-work time and that implies to disengage from job-related thoughts (Sonnentag and Bayer, 2005). This disengagement can occur rather automatically, for instance when being absorbed in another activity that requires mental presence (Hahn et al., 2012). Disengagement might also be attained by deliberate effort, for instance when following some kind of meditation practice (Michel et al., 2014). When detaching from work during non-work time, employees refrain from job-related cognitions and worries, they disengage from problem-solving attempts and planning, and they may even temporarily forget positive work events as well. Being fully detached from work versus being fully immersed in thinking about work can be seen as the two ends of a continuum.

Research has shown that people differ in the degree to which they detach from work during the evening, with high levels of job involvement and chronic job stressors being negatively related to psychological detachment from work during non-work time (Park et al., 2011; Jalonen et al., 2015). Moreover, psychological detachment also fluctuates within persons from day to day, with long working hours and negative work events during the specific day predicting low levels of psychological detachment during the evening (Sonnentag and Bayer, 2005; Bono et al., 2013).

### The Affective Benefits of Psychological Detachment From Work

Correlational research suggests that detaching from work during non-work time is related to subsequent affective states, specifically to lower levels of negative affect (Feuerhahn et al., 2014) and higher levels of positive affect (Rodríguez-Muñoz et al., 2018), as has been reflected in the meta-analysis of Wendsche and Lohmann-Haislah (2017) who showed a correlation of *r* = 0.28 between detachment and positively coded affect measures. Despite this evidence, questions about causality remain unanswered. For instance, having experienced a stressful and unpleasant day at work may increase negative affective states (Pindek et al., 2019) that in turn may make psychological detachment from work more difficult (Volmer et al., 2012) resulting in a spurious correlation between lack of psychological detachment and high negative affect. In the present research we aim at addressing this causality issue. We propose that psychologically detaching from work during non-work time has a causal impact on subsequent affective states. Specifically, a high level of psychological detachment should lead to low negative affect and to high positive affect. Negative affect is characterized by feelings of distress, fear, or anger (Watson, 1988), whereas positive affect can be described by states such as excitement, energy, alertness, and determination (Watson, 1988). We focus on state affect as rather short-term affective experiences that may change within a short period of time – as opposed to trait affect. In line with other research on affect at the interface between the work and non-work domain (Judge and Ilies, 2004), we concentrate on activated negative affect and activated positive affect.

We propose that lack of detachment from work increases negative affect and reduces positive affect – compared to overall thinking about work (i.e., negative thinking, positive thinking, unspecific thinking taken together). There are at least two reasons why lack of detachment from work should lead to negative affect and to low positive affect. First, empirical evidence suggests that when people do not detach from work, they most likely think about the negative aspects of their work (Meier et al., 2016), making it most likely that negative events and experiences remain mentally present what will lead to an increase in negative affect and a decrease in positive affect (Wang et al., 2013; Baranik et al., 2017). Second, even if both positive and negative events and experiences are mentally present when not detaching from work, negative events and experiences will have more impact on subsequent affect. Within the broader psychological literature, it has been argued that when making judgments, people weigh negative information more than positive information (Baumeister et al., 2001; Rozin and Royzman, 2001). With respect to everyday work experiences, this negativity bias implies that negative events are represented more intensely in memory than are positive events (Miron-Shatz et al., 2009). As a consequence, when not detaching from work, negative affect will increase more strongly than will positive affect – even when positive events have been experienced as well. Empirical evidence on the effects of work events points into a similar direction. For instance, negative events tend to be associated with strong negative reactions (Lim et al., 2018; Meier and Cho, 2018) whereas positive events show weaker associations with positive reactions and reduced negative reactions (Zohar et al., 2003; Bono et al., 2013). These findings imply that when people do not detach from work after having experienced both negative and positive events, it is more likely that the negative events will color the affective states resulting from not detaching from work. Accordingly, we hypothesize that – overall – low psychological detachment from work should result in high negative affect and low positive affect.

Hypothesis 1: compared to overall thinking about work, psychological detachment from work leads to (a) decreased state negative affect and (b) increased state positive affect.

### Detaching From Work Versus Various Ways of Thinking About Work

Besides this assumed overall effect of psychological detachment on subsequent affect, it is important to address the question of *how* people think about work when they do not detach from it, and if detachment is superior to all kinds of not detaching from work. For instance, when not detaching from work, people might ruminate about a problem they have encountered during the day, they might recall an episode when they received positive feedback from an important customer or they might just remember some events that are rather neutral in affective valence. Thus, sometimes work-related thoughts may have a more negative valence, sometimes they may have a more positive valence, and sometimes they may include both negative and positive aspects, or even be relatively neutral. We argue that the way employees think about their day at work has an effect on subsequent affect and shapes the contrast to psychological detachment.

#### Thinking Negatively About Work

Compared to mentally detaching from work, thinking negatively about one’s work after the end of the workday will increase state negative affect. Thinking negatively means to focus on stressful or other undesirable events that have happened at work, to focus on possible unfavorable consequences of these events, or to anticipate negative events. Thinking negatively often occurs as unconstructive repetitive thoughts (Watkins, 2008) and preseverative cognition (Lazarus, 1991) and may include rumination or worry.

The affective consequences of thinking negatively about work can be explained by appraisal theory of emotion (Lazarus, 1991; Scherer, 1999). Appraisal theory of emotion describes that cognitive evaluations of personally significant events, objects or situations elicit specific affective states. It is the way of how one thinks about an event that has a profound impact on the experienced emotion. Importantly, not only cognitive evaluations of ongoing events influence affect, also cognitive evaluations of recollected events or the mere mental presence of affective events can trigger the affective reaction (Morris, 1989; Scherer, 1999). Appraisal theory of emotion implies that work events do not only have an immediate impact on employee affect (Weiss and Cropanzano, 1996), but can also have a delayed effect when they are recollected later. For instance, remembering a stressful argument with one’s boss that has occurred during the workday and ruminating about possible negative consequences will elicit negative affect and dampen positive affect when being at home.

Empirical research demonstrates that thinking negatively about work during non-work times is indeed related to unfavorable states. For instance, negative work reflection during a vacation was associated with an increase in health problems and emotional exhaustion during the vacation period (Fritz and Sonnentag, 2006). Day-level studies by Meier et al. (2016) showed that negative work reflection during the evening was related to an increase in angry mood at bedtime and in the next morning. Similarly, Firoozabadi et al. (2018) reported that rumination about work-related troubles after work was associated with high negative affect and low positive affect in the next morning.

In contrast to thinking negatively about the day at work, psychological detachment implies not to think about work during after-work hours, neither in a negative nor in a positive way. Consequently, negative events that might have happened during the day at work are not mentally present and therefore cannot become the subject of further negative appraisal processes. Thus, although negative events might have happened during the day, they will not elicit negative affect or reduce positive affect during after-work hours. Unfavorable affective states that might have been present during the workday, will be reduced when detaching from work during the evening.

Hypothesis 2: compared to thinking about work in a negative way, psychological detachment from work leads to (a) decreased state negative affect and (b) increased state positive affect.

#### Thinking Positively About Work

In Hypothesis 1, we have argued that – overall – psychological detachment from work should lead to a decrease in state negative affect and to an increase in state positive affect. However, when contrasting psychological detachment from work with thinking positively about work, matters will be different. Thinking positively about one’s day at work will have an advantage over detaching from work and will reduce negative affect and increase positive affect. Thinking about work in a positive way means to think about desirable events at work, possible consequences of these events or to anticipate such events. For instance, one might think about a goal that one has achieved, a positive feedback from a client, or one might remember an inspiring conversation with one’s boss. Thinking positively about events has been described as a typical savoring strategy (Bryant, 2003) that helps to reduce negative affect (Hurley and Kwon, 2012) and to increase positive emotions (Quoidbach et al., 2010).

According to appraisal theory of emotion (Lazarus, 1991; Scherer, 1999), positive evaluations of events elicit positive affective states. This will not only be the case in the very situation when the event happens (Weiss and Cropanzano, 1996), but also when recollecting the event (Morris, 1989) because the positive event becomes mentally present again. Thinking in a positive way about work after the end of the workday is one prototypical approach of remembering positive events and of evaluating events in a positive way. These positive memories and evaluations will increase positive affect. Positive affective states, in addition, will help in reducing negative affective states (Fredrickson, 1998). Therefore, thinking positively about one’s work will not only boost state positive affect, but will reduce state negative affect as well.

Thinking positively about work (Sonnentag and Grant, 2012; Meier et al., 2016) as well as psychologically detaching from it (Sonnentag and Bayer, 2005; Feuerhahn et al., 2014) is associated with positive affective states. A direct comparison of thinking positively about work and detaching from work, however, is missing. Building on appraisal theory of emotion (Lazarus, 1991; Scherer, 1999), we argue that the benefit of thinking positively about work for subsequent affect should be stronger than the benefit of detaching from work. Thinking positively about work implies to stimulate positive affective states and to reduce negative states by remembering positive events and experiences and by focusing on positive appraisals. Detaching from work means to gain mental distance from both negative and positive aspects of one’s work. Detachment is powerful because it reduces the affective consequences of thinking about work. Accordingly, compared to negatively thinking about work detachment is the better option because it reduces the negative affective implications of thinking negatively about work. In situations, however, when thinking about work is dominated by positive thoughts, detaching from work would not be the best option. When detaching from the positive aspects of one’s work, one loses the opportunity to capitalize on the positive events, and consequently positive affect will not increase and negative affect will not decrease. As a consequence, when detaching from all aspects of one’s work, the resulting change in affect will be smaller than when thinking positively about one’s work. Based on this reasoning, we formulate Hypothesis 3:

Hypothesis 3: compared to thinking about work in a positive way, psychological detachment from work leads to (a) a smaller decrease in state negative affect and (b) a smaller increase in state positive affect.

#### Thinking About Work in an Unspecific Way

In daily life, there will often be a mix of positive, negative, or even rather neutral thoughts about an event. Moreover, people might not just remember one specific event, but several events with a mix of positive, negative, and relatively neutral thoughts. We call this type of thinking that might include elements of negative, positive or neutral valence *unspecific thinking*. Thus, thoughts in this category cover a broader spectrum and can be negative, positive, neutral or combinations of negative, positive, and neutral thoughts. In line with our reasoning underlying Hypothesis 1, we expect that in the case of unspecific thinking about work, negative events and experiences receive more attention than positive or neutral ones. Accordingly, negative affect should increase and positive affect should decrease.

Hypothesis 4: compared to thinking about work in an unspecific way, psychological detachment from work leads to (a) decreased state negative affect and (b) increased state positive affect.

### The Role of End-of-Work Affect

When leaving work at the end of the workday, people differ in their momentary affective states, with some experiencing elevated levels of negative affect and others experiencing elevated levels of positive affect. We propose that a person’s affective state at the end of work will be important when comparing the affective benefit of detaching from work with the affective consequences of unspecific thinking about work. Specifically, when end-of-work state negative affect is high, persons will particularly benefit from detaching from work, and when end-of-work state positive affect is high, persons will benefit from *not* detaching from work (i.e., continued thinking about work).

End-of-work affect influences how one thinks about the workday via mood-congruent information processing. Research on mood-congruent recall showed that people tend to retrieve information from memory that is congruent with their momentary affective state (Bower, 1981). More specifically, negative affect helps to recall negative information (Laird et al., 1982) and positive affect helps to recall positive information (Isen et al., 1978). In addition, momentary affect also has an effect on interpretation and judgment processes (Blanchette and Richards, 2010). For instance, when persons are in a negative (i.e., anxious or angry) state, they interpret ambiguous stimuli in a more negative (i.e., threatening) way than when they are in a positive or neutral state (Barazzone and Davey, 2009). Moreover, when they are in a negative affective state they evaluate the likelihood of future negative events as much higher than when they are in a positive affective state – and vice versa (Mayer et al., 1992).

These findings on mood-congruent recall, interpretation, and judgment suggest that a person’s affective state will influence how this person thinks about work events he or she recalls, with a higher likelihood of negative thoughts when momentary state negative affect is high and a higher likelihood of positive thoughts when momentary state positive affect is high. When not detaching from work when end-of-work negative affect is high, work-related thoughts will become particularly negative and they might appear more severe, what in turn will increase state negative affect even further (Meier et al., 2016; Firoozabadi et al., 2018). However, when not detaching from work when end-of-work positive affect is high, work-related thoughts will be more positive, what in turn will boost state positive affect (Sonnentag and Grant, 2012; Meier et al., 2016). Accordingly, end-of-work affect should moderate the benefits of psychological detachment from work. The affective benefits of psychological detachment from work should be stronger when end-of-work negative affect is high and should be weaker when end-of-work positive affect is high. Accordingly, we formulate the following hypotheses:

Hypothesis 5: when comparing psychological detachment from work with unspecific thinking about work, state negative affect after work moderates the effect of psychological detachment on subsequent negative affect. When state negative affect after work is high, the effect of psychological detachment from work on subsequent negative affect will be stronger than when state negative affect after work is low.

Hypothesis 6: When comparing psychological detachment from work with unspecific thinking about work, state positive affect after work moderates the effect of psychological detachment on subsequent positive affect. When state positive affect after work is high, the effect of psychological detachment from work on subsequent positive affect will be weaker than when state positive affect after work is low.

## Materials and Methods

We tested our hypotheses in two experimental studies. Study 1 was based on a student sample, Study 2 was based on an employee sample and aimed at replicating findings from Study 1 and at providing a more rigorous test of background variables. In both studies we contrasted two detachment conditions with three thinking-about-work conditions. We did not expect any differences between the two detachment conditions. We used two distinct detachment conditions (thinking about a hobby; being explicitly instructed to detach) in order to be better able to attribute the expected detachment effect not just to one rather specific detachment manipulation, but to a more general underlying detachment process that may have its origin in other detachment-eliciting instructions as well.

### Participants

#### Student Sample (Study 1)

Participants were 122 students at two German universities, recruited via posters and flyers distributed on campus. Participants could receive course credits or could take part in a raffle where they could win a voucher for an online retailer worth 25 Euro. The majority of the study participants were female (73.8%). Mean age was 22.6 years (*SD* = 3.6). Participants studied different majors, with the majority studying psychology (55.7%), languages and other humanities (19.6%), and sociology and political sciences (10.6%). On average, they had completed 3.9 semesters (*SD* = 3.4). Participants were randomly assigned to one of five experimental conditions.

#### Employee Sample (Study 2)

To sample employees from a broad range of jobs, we recruited participants from the local community, mainly via flyers, press releases, and advertisements in local magazines. Participants were compensated with 20 Euro for taking part in the study. A total of 163 persons participated in the study. Four persons did not comply with the instructions and were therefore excluded from the analysis. The final sample included 159 persons (61.6% female). Mean age was 36.9 years (*SD* = 10.8) and mean professional tenure was 12.3 years (*SD* = 10.5). Participants worked in a broad range of different jobs, including – among others – administrative jobs, professional jobs (e.g., IT specialist, researcher), and jobs as social workers. Mean working time per week was 37.6 h (*SD* = 5.4). On average, participants were highly educated with 60.0% having a high school (“Abitur”) or a university degree. Participants were randomly assigned to one of five experimental conditions.

### Procedure

Participants arrived at the lab for individual sessions in the late afternoon or early evening after a usual day at the university or a day on the job. Participants first provided demographic information. In addition, participants in the employee sample (Study 2) completed measures about their work situation during their day at work. Then participants from both studies completed baseline measures about their momentary affective states (state negative affect, state positive affect).

Next, a research assistant provided instructions in the five experimental conditions that should help participants to detach from their day at the university or on the job (in the two detachment conditions) or to think about it (in the three thinking conditions). This phase lasted 10–12 min in total. Participants then completed measures about momentary state affect and responded to manipulation-check items. Participants were debriefed and thanked. Before the debriefing phase, participants in the negative-thinking condition were instructed to think about a hobby (similar to instruction in the hobby condition) in order to prevent a potential spillover of negative affect into participants’ daily life.

### Experimental Conditions

Based on the literature on psychological detachment (Sonnentag and Bayer, 2005) and positive and negative work reflection (Fritz and Sonnentag, 2006), we developed manipulations for the five experimental conditions (two detachment conditions, three thinking-about-work conditions). The positive-thinking manipulation shared some similarities with positive-reflection exercises (Bono et al., 2013; Meier et al., 2016). The negative-thinking and unspecific-thinking manipulations followed the procedure of the positive-thinking manipulation with positive statements being replaced by negative ones (negative-thinking condition) or replaced by neutral ones (unspecific-thinking condition). The scripts of all experimental conditions are provided in the Supplementary Table 1. Because our hypotheses focused on differences between detachment from work versus various ways of thinking about work, we did not include a control condition (i.e., a condition without any manipulation).

In the first detachment condition (hobby condition), participants were instructed to think about a hobby. Specifically, they were told: “I would like to ask you to think about a hobby that you enjoy pursuing. If you do not have a specific hobby, you may think about another leisure activity you enjoy doing. Please remember a situation when you were engaged in your hobby or the leisure activity. Please remember what you have done or thought in this situation.” Participants were encouraged to imagine the situation and to engage themselves mentally with it. They were encouraged to take notes about the situation and their feelings in the situation. They were then instructed to think about possible consequences of engaging in this hobby or leisure activity, and to take a few notes again.

In the second detachment condition (explicit detachment instruction), participants received a directed instruction to detach. Specifically, the research assistant told: “I would like to ask you to detach from your day at the university (at your job). Please use the following minutes to think about something different. You may – if you like – just daydream a little, but without thinking about your day at the university (at your job).” In addition, participants were provided with some journals (related to fashion, politics, and sports) and were given the opportunity to browse through these journals. Participants were not allowed to read other material (e.g., material related to their job or study) or to use their smartphones.

In all three thinking-about-work conditions, the research assistant introduced the manipulation with a few sentences “I would like to ask you to think about your day at the university (at your job). Please call back to mind today’s day at the university (at your job),” before providing the specific manipulation. In the negative thinking condition, participants were instructed “Please remember in particular what did not go well, and what has stressed, upset or worried you at the university (at your job) today.” If participants could not think of a negative situation, they were instructed to extend the time frame and to think about something that did not go well during the past week and that has stressed, upset or worried them. Participants were encouraged to imagine the situation and to engage themselves mentally with it, and to take notes about the situation and their feelings in the situation. They were then instructed to think about possible negative consequences of these events or experiences, and to take a few notes again.

In the positive thinking condition and the unspecific thinking condition, the procedures were very similar, except for the positing-thinking and unspecific-thinking instructions: “Please remember in particular what did go well, what has pleased or relieved you or anything else that has put you in a positive mood at the university (at your job) today” (positive thinking) and “Please remember in particular how your day at the university (at your job) proceeded, what you have done or thought at the university (at your job) today” (unspecific thinking).

### Measures

Table 1 shows the zero-order correlations for the study variables across all conditions.

**TABLE 1:** Correlations between study variables (Study 1 and Study 2).

	**1**	**2**	**3**	**4**	**5**	**6**	**7**	**8**	**9**	**10**	**11**
Control and background variables											
(1) Quantitative demands											
(2) Organizational constraints	0.19										
(3) Perceived prosocial impact	−0.03	−0.05									
(4) Baseline negative affect	0.16	0.14	−0.10		−0.09	0.51	−0.21	0.17	−0.12	−0.3	−0.07
(5) Baseline positive affect	0.04	−0.04	0.26	−0.24		0.10	0.70	−0.09	0.12	0.02	0.01
Dependent variables											
(6) Negative affect	0.08	0.10	−0.06	0.46	−0.04		−0.13	0.60	−0.23	−0.35	−0.26
(7) Positive affect	0.07	−0.01	0.16	−0.10	0.77	−0.08		−0.30	0.13	0.27	0.33
Manipulation check											
(8) Negative thinking	0.14	0.26	−0.03	0.19	−0.17	0.41	−0.20		−0.18	−0.65	−0.51
(9) Positive thinking	−0.06	−0.06	0.25	−0.04	0.22	−0.07	0.34	−0.07		−0.38	−0.23
(10) Detachment experience	0.07	−0.04	−0.13	−0.04	0.08	−0.25	0.11	−0.52	−0.37		0.71
(11) Hobby	0.08	0.01	−0.09	0.05	0.03	−0.15	0.14	−0.39	−0.24	0.63	

*Correlations from Study 1 (*N* = 122 students) are displayed above the diagonal, with correlations of | *r*| ≥ 0.24 being significant at *p* < 0.01 and correlations of | *r*| ≥ 0.18 being significant at *p* < 0.05. Correlations from Study 2 (*N* = 159 employees) are displayed below the diagonal, with correlations of | *r*| ≥ 0.21 being significant at *p* < 0.01 and correlations of | *r*| ≥ 0.16 being significant at *p* < 0.05.*

#### Manipulation Checks

To gain information about the effectiveness of our manipulations, we asked participants to respond to a set of items on a 5-point scale (1 = *I fully disagree;* 5 = *I fully agree*), assessing negative thinking, positive thinking, detachment experience, and thinking about a hobby. The negative-thinking and positive-thinking measures were inspired by items capturing negative and positive work reflection (Fritz and Sonnentag, 2006). The detachment-experience measure was based on the psychological-detachment measure of the Recovery Experience Questionnaire (Sonnentag and Fritz, 2007). Specifically, participants answered three items assessing negative thinking (sample item: “During the past 10–15 min, I thought intensively about the negative aspects of my work,” α = 0.93 in the student sample, α = 0.94 in the employee sample), three items about positive thinking (sample item: “During the past 10–15 min, I thought intensively about the positive aspects of my work,” α = 0.94 in the student sample, α = 0.92 in the employee sample), four items about the detachment experience (sample item: “During the past 10–15 min I gained distance from the demands of my work; α = 0.92 in the student sample, α = 0.89 in the employee sample), and two self-developed items about thinking about a hobby in particular (sample item: “During the past 10–15 min I thought intensively about a hobby or a leisure activity,” *r* = 0.82 in the student sample and *r* = 0.91 in the employee sample).

#### Dependent Variables

As dependent measures, we assessed state negative and positive affect with items from the Positive and Negative Affect Schedule (PANAS; Watson et al., 1988). Participants were asked to respond to all affect items with respect to how they felt “now, in this moment,” using a five-point scale (1 = *not at all*; *5* = *extremely*). Specifically, in the student sample we used six items to assess negative affect (“distressed,” “upset,” “irritable,” “nervous,” “jittery,” and “afraid”) and six items to assess positive affect (“active,” “interested,” “excited,” “strong,” “inspired,” and “alert”). Cronbach’s alphas were 0.85 for negative affect and 0.77 for positive affect. In the employee sample, we used all ten PANAS items to assess negative affect and all ten PANAS items to assess positive affect^1^. Cronbach’s alphas were 0.90 for positive affect and 0.88 for negative affect.

#### Control Variables and Work-Situation Variables

To capture change in affect – as opposed to an absolute affect level – as recovery indicator (Zijlstra et al., 2014) we controlled for baseline state affect before the manipulations. Specifically, we assessed baseline state negative and state positive affect before the detachment and thinking-about work manipulations started, using the same set of items that we used as dependent variables. Cronbach’s alpha for negative affect were 0.72 (student sample, six items) and 0.65 (employee sample, ten items), Cronbach’s alpha for positive affect were 0.78 (student sample, six items) and 0.88 (employee sample, ten items).

To examine if participants’ workdays had been similar across the five experimental conditions, we assessed three work-situation variables in Study 2. In order to include both negative and positive experiences, we focused on quantitative demands, organizational constraints, and perceived prosocial impact. Earlier research has shown that quantitative demands (Ilies et al., 2010), organizational constraints (Rodell and Judge, 2009), and perceived prosocial impact (Sonnentag and Starzyk, 2015) are highly relevant for employee affect at work. Specifically, we assessed day-specific quantitative demands with three items based on the time-pressure measure developed by Semmer (1984; Zapf, 1993; sample item: “Today I was required to work fast”; α = 0.84), day-specific organizational constraints with three items based on a measure from Best et al. (2005; sample item: “Today, I found it difficult to do my job well because company policies restricted my efforts”; α = 0.92), and day-specific perceived prosocial impact with three items from Grant (2008; sample item: “Today I felt that my work makes a positive difference in other people’s lives”; α = 0.87). Participants responded to all items on a 5-point scale (1 = *I fully disagree;* 5 = *I fully agree*).

### Data Analysis

We analyzed our data with SPSS (Version 24).

## Results

### Manipulation Checks

#### Study 1

Analysis of variance (ANOVA) with data from the student sample showed significant main effects of the manipulations on negative thinking, *F*(4,117) = 53.462, *p* < 0.001, $\eta_{\text{p}}^{2}$ = 0.646, positive thinking, *F*(4,117) = 53.462, *p* < 0.001, $\eta_{\text{p}}^{2}$ = 0.450, detachment experience, *F*(4,117) = 76.484, *p* < 0.001, $\eta_{\text{p}}^{2}$ = 0.723, and thinking about a hobby, *F*(4,117) = 53.034, *p* < 0.001, $\eta_{\text{p}}^{2}$ = 0.645 (see Table 2 for descriptives). *Post hoc* comparisons using the Tukey HSD test are presented in the Online Supplementary Table 2. Overall, these analyses show that our manipulations were successful.

**TABLE 2:** Means and standard deviations by condition (Study 1, *N* = 122 students).

	**Explicit detachment instruction (*n* = 25)**	**Hobby condition (*n* = 24)**	**Negative thinking (*n* = 24)**	**Positive thinking (*n* = 25)**	**Unspecific thinking (*n* = 24)**
**Manipulation check**					
Negative thinking	1.33_a_ (0.51)	1.25_a_ (0.50)	4.13_d_ (0.99)	2.08_b_ (0.91)	3.07_c_ (1.03)
Positive thinking	1.75_a_ (0.80)	1.99_a_ (1.06)	1.79_a_ (0.82)	3.83_c_ (0.82)	2.78_b_ (0.99)
Detachment experience	3.55_b_ (0.98)	3.98_b_ (0.77)	1.35_a_ (0.37)	1.61_a_ (0.50)	1.70_a_ (0.67)
Hobby	3.18_b_ (0.96)	4.77_c_ (0.36)	1.75_a_ (1.05)	1.89_a_ (1.00)	1.79_a_ (0.87)
Control variables					
Baseline negative affect	1.54 (0.45)	1.34 (0.43)	1.47 (0.47)	1.55 (0.68)	1.32 (0.29)
Baseline positive affect	2.70 (0.60)	2.90 (0.42)	2.84 (0.82)	2.91 (0.64)	2.61 (0.52)
**Dependent variables**					
Negative affect	1.30 (0.29)	1.22 (0.43)	2.09 (0.92)	1.41 (0.44)	1.67 (0.75)
Positive affect	2.82 (0.55)	3.17 (0.61)	2.55 (0.80)	2.89 (0.56)	2.46 (0.53)
Single group repeated measure effect size					
Change in negative affect	−0.98	−0.58	0.96	−0.29	0.97
Change in positive affect	0.31	0.58	−0.47	0.05	−0.43

*Means that do not have the same subscripts differ at *p* < 0.05 based on Tukey’s HSD *post hoc* tests (displayed for manipulation check only).*

#### Study 2

Findings were very similar in the employee sample. Experimental conditions differed with respect to negative thinking, *F*(4,154) = 31.992, *p* < 0.001, $\eta_{\text{p}}^{2}$ = 0.449, positive thinking, *F*(4,154) = 33.229, $\eta_{\text{p}}^{2}$ = 0.463, detachment experience, *F*(4,154) = 42.972, *p* < 0.001, $\eta_{\text{p}}^{2}$ = 0.527, and thinking about a hobby, *F*(4,154) = 76.995, *p* < 0.001, $\eta_{\text{p}}^{2}$ = 0.667 (see Table 3 for descriptives). *Post hoc* comparisons using the Tukey HSD test are displayed in the Online Supplementary Table 3. Again, these analyses demonstrate that our manipulations were successful.

**TABLE 3:** Means and standard deviations by condition (Study 2, *N* = 159 employees).

	**Explicit detachment instruction (*n* = 32)**	**Hobby condition (*n* = 33)**	**Negative thinking (*n* = 32)**	**Positive thinking (*n* = 31)**	**Unspecific thinking (*n* = 31)**
**Manipulation check**					
Negative thinking	1.28_a_ (0.66)	1.97_a,b_ (1.01)	3.93_d_ (0.99)	2.10_b,c_ (1.17)	2.77_c_ (1.14)
Positive thinking	1.48_a_ (0.80)	2.52_b_ (1.18)	2.19_b_ (0.89)	4.16_d_ (1.03)	3.22_c_ (1.02)
Detachment experience	3.80_b_ (1.03)	3.39_b_ (1.19)	1.74_a_ (0.66)	1.55_a_ (0.71)	1.82_a_ (0.80)
Hobby	2.89_b_ (1.32)	4.44_c_ (0.83)	1.17_a_ (0.57)	1.40_a_ (0.93)	1.40_a_ (0.61)
Control and background variables					
Quantitative demands	2.68 (1.08)	2.49 (1.19)	2.62 (1.12)	2.57 (1.17)	2.46 (0.98)
Organizational constraints	1.64 (0.83)	1.76 (1.01)	1.69 (0.83)	1.63 (1.17)	1.63 (0.85)
Perceived prosocial impact	3.35 (1.08)	3.81 (1.12)	3.58 (1.03)	3.54 (1.25)	3.54 (0.88)
Baseline negative affect	1.22 (0.21)	1.22 (0.21)	1.12 (0.18)	1.25 (0.33)	1.28 (0.36)
Baseline positive affect	3.11 (0.77)	3.04 (0.80)	3.08 (0.60)	2.89 (0.79)	2.95 (0.67)
Dependent variables					
Negative affect	1.05 (0.08)	1.15 (0.20)	1.30 (0.35)	1.15 (0.25)	1.36 (0.62)
Positive affect	2.95 (0.85)	3.27 (0.72)	3.01 (0.60)	3.22(0.80)	2.79 (0.72)
Single group repeated measure effect size					
Change in negative affect	−1.21	−0.28	0.58	−0.53	0.18
Change in positive affect	−0.38	0.44	−0.21	0.52	−0.40

*Means that do not have the same subscripts differ at *p* < 0.05 based on Tukey’s HSD *post hoc* tests (displayed for manipulation check only).*

### Test of Hypotheses: Overall Effects

First, we tested an overall effect of our manipulations, using multivariate analysis of covariance (MANCOVA) with post-manipulation negative and positive affect as dependent variables, the five experimental conditions as independent variables, and baseline (i.e., pre-manipulation) negative and positive affect as control variables. The overall multivariate effect was significant in the student sample, *F*(8,230) = 7.750, *p* < 0.001, Pillai Trace = 0.425, $\eta_{\text{p}}^{2}$ = 0.21, and in the employee sample, *F*(8,304) = 5.878, *p* < 0.001, Pillai Trace = 0.268, $\eta_{\text{p}}^{2}$ = 0.13. Also univariate tests were significant for negative affect in the student sample, *F*(4,115) = 13.917, *p* < 0.001, $\eta_{\text{p}}^{2}$ = 0.33, and in the employee sample, *F*(4,152) = 6.249, *p* < 0.001, $\eta_{\text{p}}^{2}$ = 0.14. Similarly, univariate tests were significant for positive affect in the student sample, *F*(4,115) = 6.897, *p* < 0.001, $\eta_{\text{p}}^{2}$ = 0.19, and in the employee sample, *F*(4,152) = 5.532, *p* < 0.001, $\eta_{\text{p}}^{2}$ = 0.13. Tables 2, 3 show means and standard deviations of our dependent variables in the two samples. These MANCOVA results provide first evidence that the manipulations had an effect on participants’ affect.

### Test of Hypotheses: Differences Between the Five Experimental Conditions

We then tested our specific hypotheses with a set of multiple regression analyses, using contrast coding as described by Cohen et al. (2003). Specifically, we built five contrast codes: the two detachment conditions versus the three thinking-about-work conditions taken together, the two detachment conditions versus negative thinking, the two detachment conditions versus positive thinking, the two detachment conditions versus unspecific thinking, and explicit detachment instruction versus thinking about a hobby. Table 4 shows how the effects were coded. We included baseline negative affect as a control variable when predicting negative affect and baseline positive affect when predicting positive affect^2^ (Model 1). We tested the overall effect of detachment (i.e., explicit detachment condition and hobby condition considered together by coding both conditions in the same way) versus overall thinking about work in Model 2a (Hypothesis 1). In Model 2b, we tested the effect of detachment versus the three separate thinking-about-work conditions (Hypotheses 2, 3, and 4). For testing Hypotheses 5 and 6, we entered the interaction terms between baseline affect and the code for unspecific thinking (Model 3) and tested this model against Model 2b. We evaluated model fit by computing *R*^2^ and Δ*R*^2^.

**TABLE 4:** Coding of contrasts for five experimental conditions.

	**Explicit detachment instruction**	**Hobby condition**	**Negative thinking**	**Positive thinking**	**Unspecific thinking**
Two detachment conditions versus three thinking conditions	1/2	1/2	−1/3	−1/3	−1/3
Difference between two detachment conditions (explicit detachment instruction versus hobby condition)	1/2	−1/2	0	0	0
Two detachment conditions versus negative thinking	1/3	1/3	−2/3	0	0
Two detachment conditions versus positive thinking	1/3	1/3	0	−2/3	0
Two detachment conditions versus unspecific thinking	1/3	1/3	0	0	-2/3

*Entries show how the experimental conditions were coded in the regression analyses.*

#### Study 1

Table 5 shows the findings for Study 1. For negative affect as dependent variable, baseline negative affect as control variable was a strong predictor in Model 1. When entering the contrast between the two detachment conditions versus all three thinking-about-work conditions together, in addition to the contrast between the two detachment conditions, model fit improved (Model 2a). Compared to thinking about work, detachment reduced negative affect, providing support for Hypothesis 1a.

**TABLE 5:** Findings from ordinary least square regression analysis (Study 1).

	**Negative affect**					**Positive affect**				
	***b***	***SE***	**β**	***t***		***b***		***SE***	**β**	***t***
**Model 1**										
Intercept	0.513	0.167		3.062**	0.710		0.199		3.564**	
Baseline negative affect	0.708	0.110	0.507	6.441***	—					
Baseline positive affect	—				0.740		0.070	0.696	10.623***	
*R*^2^	0.257					0.485				
*F*	41.487***				112.856***					
**Model 2a**										
Intercept	0.514	0.157		3.269**	0.746		0.186		4.002***	
Baseline negative affect	0.708	0.103	0.507	6.850***	—					
Baseline positive affect	—				0.727		0.065	0.684	11.170***	
Explicit detachment instruction versus hobby condition^a^	−0.059	0.157	−0.028	−0.374		−0.199		0.127	−0.096	−1.573
Detachment versus thinking about work^b^	−0.539	0.121	−0.327	−4.454***	0.421		0.098	0.262	4.307***	
*R*^2^	0.365					0.562				
*F*	22.591***				50.448***					
Δ*R*^2^ (compared to Model 1)	0.108					0.077				
*F*	10.023***				10.402***					
**Model 2b**										
Intercept	0.473	0.144		3.283**	0.777		0.185		4.188***	
Baseline negative affect	0.738	0.095	0.528	7.772***	—					
Baseline positive affect	—				0.716		0.065	0.673	11.032***	
Explicit detachment instruction versus hobby condition^a^	−0.065	0.143	−0.030	−0.453		−0.202		0.124	−0.098	−1.619
Detachment versus negative thinking^c^	−0.788	0.135	−0.426	−5.819***	0.399		0.119	0.221	3.363**	
Detachment versus positive thinking^d^	0.312	0.134	0.171	2.324*	−0.044		0.117	−0.025	−0.374	
Detachment versus unspecific thinking^e^	−0.340	0.137	−0.184	−2.491*	0.281		0.120	0.156	2.343*	
*R*^2^	0.485					0.584				
F	21.860***				32.534***					
Δ*R*^2^ (compared to Model 1)	0.228					0.099				
*F*	12.855***				6.902***					
**Model 3**										
Intercept	0.399	0.143		2.791**	0.777		0.186		4.174***	
Baseline negative affect	0.800	0.095	0.573	8.417***	—					
Baseline positive affect	—				0.716		0.065	0.673	10.987***	
Explicit detachment instruction versus hobby condition^a^	−0.013	0.140	−0.006	−0.092		−0.204		0.126	−0.099	−1.621
Detachment versus negative thinking^c^	−0.762	0.132	−0.411	−5.765***	0.400		0.119	0.222	3.535**	
Detachment versus positive thinking^d^	0.345	0.131	0.189	2.630*	−0.042		0.118	−0.024	−0.360	
Detachment versus unspecific thinking^e^	0.980	0.497	0.529	1.972		0.377		0.574	0.209	0.657
Baseline negative affect × unspecific thinking	−0.992	0.360	−0.752	−2.757**	—					
Baseline positive affect × unspecific thinking	—					−0.037		0.212	−0.055	−0.172
*R*^2^	0.517					0.584				
*F*	20.520***				26.890***					
Δ*R*^2^ (compared to Model 2b)	0.032					0.000				
F	7.602**				0.030					

*p < 0.05. **p < 0.01. ***p < 0.001. ^*a*^Explicit detachment instruction coded as 0.5, hobby condition coded as −0.5, and all other conditions coded as 0. ^*b*^Explicit detachment instruction coded as 0.5, hobby condition coded as 0.5, negative thinking coded as −0.333, positive thinking coded as −0.333, and unspecific thinking coded as −0.333. ^*c*^Explicit detachment instruction coded as 0.333, hobby condition coded as 0.333, negative thinking coded as 0.667, other conditions coded as 0. ^*d*^Explicit detachment instruction coded as 0.333, hobby condition coded as 0.333, positive thinking coded as 0.667, other conditions coded as 0. ^*e*^Explicit detachment instruction coded as 0.333, hobby condition coded as 0.333, unspecific thinking coded as 0.667, other conditions coded as 0.

In Model 2b, we tested detachment against the three separate thinking conditions (instead of the overall effect of thinking about work). Compared to Model 1, model fit improved. The contrasts between detachment and negative thinking, between detachment and positive thinking as well as between detachment and unspecific thinking were significant. Compared to the negative-thinking and the unspecific-thinking condition, negative affect decreased in the detachment conditions. Compared to the positive-thinking condition, however, negative affect increased in the detachment conditions. Table 2 shows the repeated-measures *d* effect sizes, separately for all five conditions. These effect sizes describe the change in affect from baseline to post-manipulation, taking into account that the measures of negative (positive) affect at baseline and negative (positive) affect after the manipulations are correlated (Morris and DeShon, 2002). It can be seen that negative affect increased substantially in the negative-thinking (*d* = 0.961) and unspecific-thinking (*d* = 0.967) conditions, and decreased substantially when detaching from work, particularly in the explicit-detachment condition (*d* = -0.982). Overall, this pattern of findings provides support for Hypotheses 2a, 3a, and 4a.

**FIGURE 1:**
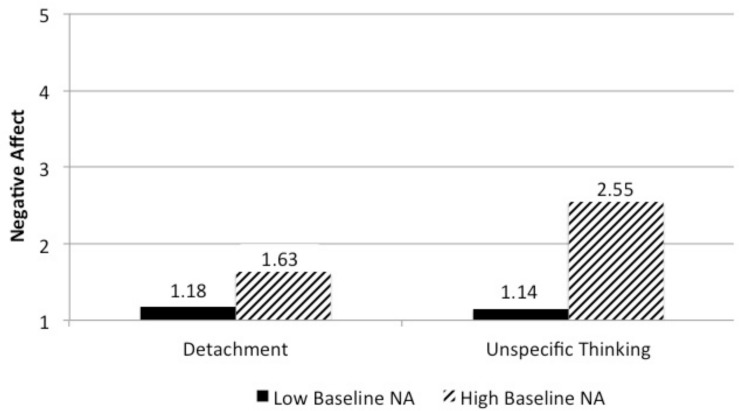
Interaction effect between baseline negative affect and unspecific thinking (Study 1).

When entering the interaction effect between baseline negative affect and unspecific thinking into the model (Model 3), model fit further improved and the interaction effect between baseline negative affect and the contrast between detachment versus unspecific thinking was significant. Because of the high multicollinearity between the two interaction effects, in an additional analysis we only entered the interaction term with baseline negative affect into Model 3. Results did not change and the interaction effect between baseline negative affect and the contrast between detachment versus unspecific thinking was significant. To examine the pattern of the significant interaction effect, we followed the approach of Preacher et al. (2006) and tested if the contrast between the two detachment conditions and unspecific thinking differed between participants with high versus low baseline negative affect. For persons with high baseline negative affect (1 *SD* above the mean), the contrast was significant, *b* = −0.945, *SE* = 0.242, *t* = −3.902, *p* < 0.001, whereas for persons with low baseline negative affect (1 *SD* below the mean) the contrast was not significant, *b* = −0.047, *SE* = 0.186, *t* = −0.251, *p* = 0.803. Figure 1 shows negative-affect scores for high versus low baseline negative affect in the two detachment conditions and the unspecific-thinking condition. When baseline negative affect was high, negative affect increased more in the unspecific-thinking condition than in the detachment conditions. No such difference was observed when baseline negative affect was low. This pattern supports Hypothesis 5a. Hypothesis 6a was not supported.

For positive affect as dependent variable, baseline positive affect as control variable was a strong predictor (Model 1). When entering the contrast between the two detachment conditions versus all three thinking-about-work conditions together, model fit improved (Model 2a). Compared to thinking about work, detachment resulted in an increase in positive affect. This finding is in line with Hypothesis 1b.

Entering contrasts between detachment and the three separate thinking conditions into Model 2b resulted in an improved fit over Model 1. The contrasts between detachment and negative thinking as well as detachment and unspecific thinking were significant. Compared to negative and unspecific thinking, detachment resulted in a larger increase in positive affect. The contrast between the two detachment conditions and positive thinking was not significant. In Model 3, the interaction terms were not significant. Overall, findings provide support for Hypotheses 2b and 4b, but neither for Hypothesis 3b nor for Hypotheses 5b or 6b.

#### Study 2

Table 6 shows the findings for the employee sample. Again, the control variable negative affect at baseline was a strong predictor of negative affect (Model 1). Entering the contrast between detachment versus all three thinking-about-work conditions together, along with the contrast between the two detachment conditions, resulted in an improved model fit (Model 2a). Compared to thinking about work, detachment from work resulted in a decrease in negative affect, supporting Hypothesis 1a.

**TABLE 6:** Findings from ordinary least square regression analysis (Study 2).

	**Negative affect**				**Positive affect**			
	***b***	***SE***	**β**	***t***	***b***	***SE***	**β**	***t***
**Model 1**								
Intercept	0.439	0.120		3.659***	0.703	0.163		4.322***
Baseline negative affect	0.625	0.096	0.461	6.501***	—			
Baseline positive affect	—				0.790	0.053	0.769	15.055***
*R*^2^	0.212				0.588			
*F*	42.257***				226.664***			
**Model 2a**								
	0.433	0.116		3.741***	0.680	0.159		4.281***
Baseline negative affect	0.631	0.093	0.464	6.786***	—			
Baseline positive affect	—				0.798	0.051	0.776	15.572***
Explicit detachment instruction versus hobby condition^a^	−0.093	0.077	−0.082	−1.202	−0.376	0.116	−0.162	−3.251**
Detachment versus thinking about work^b^	−0.208	0.060	−0.236	−3.446***	−0.048	0.090	−0.027	−0.535
*R*^2^	0.274				0.617			
*F*	19.502***				83.396***			
Δ*R*^2^ (compared to Model 1)	0.062***				0.027			
*F*	6.613**				5.404**			
**Model 2b**								
Intercept	0.396	0.115		3.435**	0.645	0.155		4.165***
Baseline negative affect	0.661	0.092	0.487	7.147***	—			
Baseline positive affect	—				0.810	0.050	0.788	16.210***
Explicit detachment instruction versus hobby condition^a^	−0.093	0.075	−0.082	−1.234	−0.377	0.112	−0.161	−3.355**
Detachment versus negative thinking^c^	−0.243	0.073	−0.247	−3.305**	0.188	0.108	0.093	1.746
Detachment versus positive thinking^d^	0.110	0.073	0.111	1.509	−0.354	0.109	−0.173	−3.243**
Detachment versus unspecific thinking^e^	−0.178	0.073	−0.179	−2.429*	0.089	0.109	0.043	0.814
*R*^2^	0.321				0.644			
*F*	14.456***				55.263***			
Δ*R*^2^ (compared to Model 1)	0.109				0.053			
*F*	6.126***				5.670***			
**Model 3**								
Intercept	0.532	0.118		4.507***	0.637	0.156		4.081***
Baseline negative affect	0.545	0.095	0.401	5.712***	—			
Baseline positive affect	—				0.813	0.051	0.790	16.088***
Explicit detachment instruction versus hobby condition^a^	−0.094	0.073	−0.083	−1.292	−0.376	0.113	−0.161	−3.332**
Detachment versus negative thinking^c^	−0.234	0.071	−0.237	−3.292**	0.190	0.108	0.094	1.757
Detachment versus positive thinking^d^	0.096	0.071	0.097	1.362	−0.353	0.110	−0.172	−3.224**
Detachment versus unspecific thinking^e^	0.790	0.288	0.794	2.745**	0.271	0.437	0.132	0.622
Baseline negative affect × unspecific thinking	−0.771	0.222	−1.007	−3.472***	—			
Baseline positive affect × unspecific thinking	—				−0.062	0.142	−0.092	−0.432
*R*^2^	0.371				0.644			
*F*	14.927***				45.839***			
Δ*R*^2^ (compared to Model 2b)	0.050				0.000			
*F*	12.956**				0.187			

*p < 0.05. **p < 0.01. ***p < 0.001. ^*a*^Explicit detachment instruction coded as 0.5, hobby condition coded as −0.5, and all other conditions coded as 0. ^*b*^Explicit detachment instruction coded as 0.5, hobby condition coded as 0.5, negative thinking coded as −0.333, positive thinking coded as −0.333, and unspecific thinking coded as −0.333. ^*c*^Explicit detachment instruction coded as 0.333, hobby condition coded as 0.333, negative thinking coded as 0.667, other conditions coded as 0. ^*d*^Explicit detachment instruction coded as 0.333, hobby condition coded as 0.333, positive thinking coded as 0.667, other conditions coded as 0. ^*e*^Explicit detachment instruction coded as 0.333, hobby condition coded as 0.333, unspecific thinking coded as 0.667, other conditions coded as 0.

Contrasts between detachment and the three separate thinking-about-work conditions entered into Model 2b contributed to an improvement of model fit over Model 1. The contrasts between detachment and negative thinking as well as between detachment and unspecific thinking were significant, whereas the contrast between detachment and positive thinking was not. Table 3 shows that negative affect increased moderately in the negative-thinking condition (*d* = 0.581), whereas it decreased substantially in the explicit-detachment condition (*d* = −1.207). The decrease in negative affect in the hobby condition was relatively small (*d* = −0.278). Taking together, these findings provide support for Hypotheses 2a and 4a.

**FIGURE 2:**
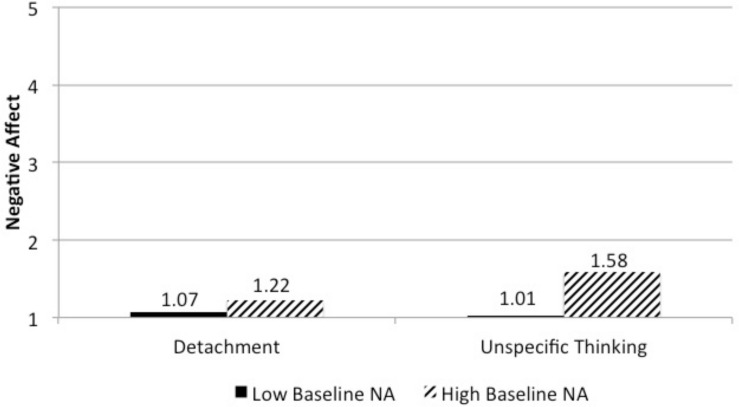
Interaction effect between baseline negative affect and unspecific thinking (Study 2).

The interaction term between baseline negative affect and the contrast between detachment versus unspecific thinking entered into Model 3 was significant, also when only entering the interaction effect between baseline negative affect and the contrast between detachment versus unspecific thinking into the model. For persons with high baseline negative affect (1 *SD* above the mean), the contrast was significant, *b* = −0.350, *SE* = 0.102, *t* = −3.438, *p* < 0.001, but for persons with low baseline negative affect (1 *SD* below the mean) it was not, *b* = 0.048, *SE* = 0.106, *t* = 0.447, *p* = 0.656. Figure 2 shows negative-affect scores, dependent on baseline negative affect and the contrast between the two detachment conditions and the unspecific-thinking condition. This pattern of findings provides support for Hypothesis 5a.

In the regression analysis for positive affect as dependent variable, baseline positive affect as control variable was a strong predictor. Model 2a including the contrast between detachment versus all three thinking conditions together as well as the contrast between the two detachment conditions showed a better model fit than Model 1. In this model, however, the contrast between detachment and overall thinking was not significant. Thinking about the hobby led to higher increase in positive affect than being explicitly instructed to detach.

Entering contrasts for the three thinking conditions separately into the model (Model 2b), resulted in an improved fit over Model 1. The contrast between the two detachment conditions and positive thinking was significant. Again, the contrast referring to differences between the two detachment conditions was significant. Positive thinking about work resulted in an increase in positive affect (*d* = 0.522), thinking about a hobby led to an increase in positive affect as well (*d* = 0.440), whereas explicitly detaching from work led to a decrease in positive affect (*d* = −0.383). Thus, the difference between positive thinking and detachment is mainly due to a detrimental effect of detachment on positive affect, thinking about a hobby resulted in an increase in positive affect. The interaction terms between baseline affect and unspecific thinking were not significant. Overall, with respect to positive affect as dependent variable, Study 2 provided support for Hypothesis 3b.

### Equivalence of Day-Specific Work Situation Across Experimental Conditions

The way of how employees have experienced their workday may impact on how they think about it afterward. To rule out that workday experiences have influenced the dependent variables, we used data from Study 2 and examined if day-specific work-situation variables (quantitative demands, organizational constraints, perceived prosocial impact) differed between the experimental conditions. A multivariate analysis of variance (MANOVA) with quantitative demands, organizational constraints, and perceived prosocial impact as dependent variables did not reveal any significant differences between the five experimental conditions, *F*(12,462) = 0.385, *ns*, Pillai Trace = 0.030, $\eta_{\text{p}}^{2}$ = 0.01. Also univariate tests were non-significant for quantitative demands, *F*(4,154) = 0.205, ns, $\eta_{\text{p}}^{2}$ = 0.01, organizational constraints, *F*(4,154) = 0.099, *ns*, $\eta_{\text{p}}^{2}$ = 0.00, and perceived prosocial impact, *F*(4,154) = 0.764, *ns*, $\eta_{\text{p}}^{2}$ = 0.02 (see Table 3 for descriptives). This analysis suggests that employees’ workday experiences did not differ across the experimental conditions.

## Discussion

Using an experimental design, our studies showed that compared to negative or unspecific thinking about work detachment from work led to more favorable affective states. Positively thinking about work, however, tended to outperform psychological detachment: In one of the studies (student sample) thinking positively about work caused a stronger decrease in state negative affect than did psychological detachment; in the other study (employee sample), thinking positively about work caused a stronger increase in state positive affect than did psychological detachment.

### Theoretical Implications

Our research makes contributions to the detachment literature and suggests that theorizing on psychological detachment from work should be refined. First, our studies show that a differentiated view on psychological detachment is needed. Although we found an overall beneficial effect of psychological detachment from work on subsequent affect, our findings further suggest that the affective valence of work-related thoughts during after-work hours plays a key role for the affective consequences of a lack of detachment from work. It seems that mainly negatively toned thoughts about work drive the detrimental effect of not detaching from work. Specifically, analyses showed that detaching from work leads to lower levels of negative affect (in both studies) and to higher levels of positive affect (in Study 1) than thinking about work in a negative or in an unspecified way. Importantly, the effect of unspecific thinking about work on negative effect was qualified by an interaction effect with baseline negative affect (i.e., end-of-work negative affect): At low levels of baseline negative affect, unspecific thinking about work did not lead to an increase in negative affect, implying that detachment from work is particularly beneficial when end-of-work negative affect is high. Overall, our findings suggest that not detaching is particularly detrimental for subsequent affect when comparing it with thinking about work dominated by negative thoughts – as was the case in the negative-thinking condition – or when end-of-work negative affect is high and when, consequently, thinking about work might be more negative – as was the case in the unspecific-thinking condition.

With respect to refining theory on psychological detachment from work, it is important to note that psychological detachment was not more beneficial for subsequent affect than thinking positively about work. In two out of four comparisons, thinking positively about work had an even more favorable effect on subsequent affect than detachment from work. Thus, positive thoughts about work can outperform the affective benefits of detaching from work. This finding supports our interpretation that the affective valence of work-related thoughts is highly relevant for explaining why lack of detachment from work has a detrimental effect on subsequent affect. Taken together, our research provides an important step in arriving at a more differentiated picture of the detrimental effects of lack of psychological detachment from work. It demonstrates that it should not be taken for granted that the most favorable affective states can be achieved by detaching from work during after-work hours. Accordingly, the predominantly positive view on psychological detachment needs to be revisited.

As a second important contribution, our studies demonstrate the causal effect of detachment from work on subsequent affect. This effect had been implied in many of the earlier studies on recovery in general (Bennett et al., 2018) and on psychological detachment in particular (Sonnentag and Fritz, 2015), but it could not be adequately addressed in correlational studies. Our findings suggest that the associations between detachment and subsequent affective states as found in previous research cannot be fully explained by third variables such as events that have happened during the day or end-of-work state affect. Instead, psychological detachment from work causes changes in affect.

Our results highlight the importance of detachment from work for affect regulation at the work-home interface. More specifically, earlier studies have shown that affect experienced at work spills over into non-work life (Ilies et al., 2007). Our findings suggest that deliberate detachment from work can be an effective approach to stop this affect-spillover process. Bringing affect-spillover to a halt will be particularly desirable when negative affect at the end of the workday is high. When positive affect at the end of the workday is high, however, detachment from work will be less beneficial for subsequent affect.

### The Role of Positive Affect as Outcome Variable

In contrast to the findings on negative affect as outcome variable, results are a bit less clear for positive affect. Thinking positively increased positive affect in the employee sample, but not in the student sample. This finding has to be seen in the light that in the student sample thinking positively reduced negative affect. Thus, when instructed to think positively about the past day at the university, students might have thought about the absence of negative experiences and events, but not about explicit positive ones. Accordingly, they might have experienced a change in negative affect, but not in positive affect.

We did not find a significant interaction effect between positive affect before the manipulation and unspecific thinking. End-of-work positive affect probably does not provide a particular strong boost in positive affect when thinking about work, as compared to detaching from it. Participants with a high baseline positive affect also might have had highly positive non-work thoughts when detaching from work or when thinking about their hobbies. Thus, when experiencing a high positive affect at the end of the workday, not only work-related thoughts might become more positive, but non-work thoughts as well.

### Detachment Manipulations

In our studies, we manipulated psychological detachment from work with two distinct instructions, the one providing an explicit detachment instruction, and the other inviting participants to think about their hobby. Out of the four contrasts tested, only one was significant: In the employee sample, positive affect increased more in the hobby condition than in the explicit-detachment condition. This pattern of finding suggests that – overall– effects on detachment can be attained by both an explicit detachment instruction and by thinking about a hobby, leading to a reduction in negative affect. When it comes to a potential increase in positive affect, the hobby instruction seems to be more successful, possibly because thinking about a hobby triggers positive thoughts in most people, whereas detaching from work does not necessarily stimulate positive thoughts. For instance, participants in the explicit-detachment condition might have directed their attention to family related problems that could have reduced positive affect.

### Limitations and Directions for Future Research^3^

Our research has some limitations. First, we used two quite distinct manipulations of psychological detachment from work. Whereas the instruction to think about a hobby provided a clear guideline about what to think about, the explicit detachment instruction was relatively vague with respect to what participants should think about or what they should do during the experimental session. Thus, we have only little insight into the specific content of the thought processes participants might have been engaged in while detaching from work. For instance, thinking about financial problems or interpersonal conflicts at home versus thinking about a relaxing weekend or the last success of one’s favorite soccer team most likely result in quite distinct affective outcomes. Future studies might want to use a broader range of detachment manipulations in order to find out what makes the detachment process most effective.

Second, we did not manipulate baseline affect. This feature of our study implies that some ambiguities remain if it was baseline affect – and not another variable associated with baseline affect – that caused the specific reaction patterns to the detachment instructions versus the thinking-about-work instruction. Future studies may want to directly manipulate baseline affect in order to rule out that more stable between-person differences or specific negative work events have driven our findings. However, such an effort would result in a rather complex experimental procedure in which first state affect and then detachment from work versus thinking about work would need to be manipulated.

Third, in our study we did not include any control condition in which participants did not receive any manipulation. Of course, it would have been interesting to compare negative and positive affect after our five manipulations with negative and positive affect after not having received any manipulation. In addition to the fact that this was not the focus of study, findings from any “empty” manipulation would be difficult to interpret in a laboratory setting. For instance, having participants just wait for a while or having participants to complete arbitrary tasks might have its own impact on affect – irrespective of the effect of not detaching from work or not thinking about work.

Finally, we examined if workday experiences differed between the various experimental groups in the employee sample only. Strictly speaking, we do not know if there were systematic differences in pre-manipulation experiences between the various experimental groups in the student sample. However, because we used random assignments, the likelihood of systematic pre-manipulation differences between the experimental groups should be relatively low.

Although our studies provide important insights into the effects of detachment from work versus thinking about work, some questions remain unanswered. First, our thinking-about-work manipulation did not address any specific work content. Ohly and Schmitt (2015) have argued that specific events experienced at work are associated with subsequent affect in a particular way. For instance, based on a literature review Ohly and Schmitt demonstrated that social conflicts (i.e., negative interpersonal events) are more strongly associated with anger (i.e., a negative affective state) than hindrances in goal attainment (i.e., a negative task-related event). Similarly, also thinking about interpersonal versus task-related events might have distinct consequences for subsequent affect. Therefore, future studies might want to use more differentiated instructions for thinking about work. In addition, one could instruct participants to think about an event that has elicited specific discrete emotions (e.g., anger versus anxiety) and examine how this influences subsequent affect. Moreover, also the intensity of the thoughts and the depth of information processing when thinking about work might be important here.

Second, although our studies suggest that the detachment instructions helped study participants to detach rather easily from work, there might be instances when psychological detachment from work cannot be easily achieved but requires effortful emotion regulation. It would be interesting to examine for whom and when psychological detachment becomes an effortful endeavor. Third, our studies used a relatively short time frame when testing the affective benefits of detaching from work and of thinking positively about work. Future studies might want to examine if the benefits continue throughout the evening – and eventually until the next day. We are aware that from a research ethics perspective some adjustments in the study procedure and particularly in the negative-thinking condition would be needed. Finally, in our two studies participants were highly educated and their thinking-about work cognitions might have reflected mentally demanding task requirements. Future studies might want to examine detachment from work and thinking about work in less well-educated samples that are facing different task requirements and working conditions.

### Practical Implications

Our findings suggest that employees’ affect during evening hours can be influenced in a favorable way by encouraging employees to think positively about their workday, to explicitly detach from work, and to direct their attention to a hobby. Our study has demonstrated that the time that is needed to change affect is relatively short: in our experimental procedure the total time of thinking about work (or detaching from it) was 10–12 min. We assume that many people can make time for these 12 min after work that they could deliberately use for reflecting positively about their workday or for intentionally switching off from work. Employees with a busy family life might consider using some time during the commute in a train or bus for this detachment process. Within the broader context of positive psychological interventions, our findings are in line with research on gratitude and capitalization interventions (Ilies et al., 2011; Davis et al., 2016). In order to think positively about work, employees may engage in short gratitude and capitalization exercises focusing on positive work events and experiences; in order to detach from work they may engage in short gratitude and capitalization exercises focusing on positive non-work events and experiences (e.g., hobbies).

Of course, not all workdays are solely positive. From time to time, employees will experience negative events at work. And particularly on those days it might be difficult to detach from work (Wang et al., 2013). Research on emotion regulation suggests that cognitive reappraisal (i.e., reinterpreting the meaning of a negative event) can influence the affective reaction to it (Ray et al., 2008). Accordingly, when immediate detachment seems impossible, employees may want to start with a reappraisal process, for instance, by seeing a negative event from the perspective of a third person.

Although our experimental procedure resulted in beneficial affective outcomes, we believe that a rather strong situation is needed in order to achieve these effects. For instance, during our experimental sessions, some participants commented that it was difficult to detach from work. Thus, in daily life employees may find it difficult to refrain from thinking about work – even when they wish to do. Possibly, building a strong habit can help to detach from work when being at home (Wood and Rünger, 2016). Strong contextual cues will support habit formation (Neal et al., 2012). For instance, employees may link a specific location (e.g., the bus stop when waiting for the bus to get home after work) as the contextual cue to detach from work (cf. Ashforth et al., 2000).

## Conclusion

Taken together, our studies showed that a differentiated perspective on psychological detachment is needed. The affective valence of work-related thoughts matters for the affective outcomes of thinking versus detaching about work. Although there is an overall affective benefit of detaching from work, explicit positive thinking about work does not result in a decline of favorable affective states.

## Data Availability Statement

The raw data supporting the conclusions of this article will be made available by the authors, without undue reservation.

## Ethics Statement

The studies involving human participants were reviewed and approved by Ethics committee of University of Mannheim. The participants provided their written informed consent to participate in this study.

## Author Contributions

SS conceived the study, supervised the data collection, analyzed the data, and wrote the manuscript. CN contributed to the study design, provided the detailed feedback on the analyses and the manuscript. Both authors contributed to the article and approved the submitted version.

## Conflict of Interest

The authors declare that the research was conducted in the absence of any commercial or financial relationships that could be construed as a potential conflict of interest.

## Footnotes

1 When performing the analyses with the six negative-affect and the six positive-affect items used in Study 1, findings did not change.

2 Not including baseline negative affect and baseline positive affect as control variables did not change the findings. Including not only the affect-symmetric baseline scores [i.e., (1) baseline negative affect when predicting negative affect as outcome variable and (2) baseline positive affect when predicting positive affect as outcome variable] but also the affect-asymmetric baseline scores [i.e., (1) baseline negative affect *and* baseline positive affect when predicting negative affect as outcome variable and (2) baseline positive affect *and* baseline negative affect when predicting positive affect as outcome variable] left the findings unchanged as well.

3 We are grateful for the comments of the two reviewers who elaborated on some of the limitations discussed here and who suggested some of the directions for future research.

## References

[B1] Ashforth B. E. Kreiner G. E. Fugate M. (2000). All in a day’s work: boundaries and micro role transitions. *Acad. Manag. Rev.* 25 472–491. 10.5465/AMR.2000.3363315

[B2] Baranik L. E. Wang M. Gong Y. Shi J. (2017). Customer mistreatment, employee health, and job performance: cognitive rumination and social sharing as mediating mechanisms. *J. Manag.* 43 1261–1282. 10.1177/0149206314550995

[B3] Barazzone N. Davey G. C. L. (2009). Anger potentiates the reporting of threatening interpretations: an experimental study. *J. Anxiety Disord.* 23 489–495. 10.1016/j.janxdis.2008.10.007 19070989

[B4] Baumeister R. F. Bratslavsky E. Finkenauer C. Vohs K. D. (2001). Bad is stronger than good. *Rev. Gen. Psychol.* 5 323–370. 10.1037/1089-2680.5.4.323

[B5] Bennett A. A. Bakker A. B. Field J. G. (2018). Recovery from work-related effort: a meta-analysis. *J. Organ. Behav.* 39 262–275. 10.1002/job.2217

[B6] Best R. G. Stapelton L. M. Downey R. G. (2005). Core self-evaluations and job burnout: the test of alternative models. *J. Occup. Health Psychol.* 10 441–451. 10.1037/1076-8998.10.4.441 16248691

[B7] Blanchette I. Richards A. (2010). The influence of affect on higher level cognition: a review of research on interpretation, judgement, decision making and reasoning. *Cogn. Emot.* 24 561–595. 10.1080/02699930903132496

[B8] Bono J. E. Glomb T. M. Shen W. Kim E. Koch A. J. (2013). Building positive resources: effects of positive events and positive reflection on work-stress and health. *Acad. Manag. J.* 56 1601–1627. 10.5465/amj.2011.0272

[B9] Bower G. H. (1981). Mood and memory. *Am. Psychol.* 36 129–148. 10.1037/0003-066X.36.2.129 7224324

[B10] Brosschot J. F. Gerin W. Thayer J. F. (2006). The perseverative cognition hypothesis: a review of worry, prolonged stress-related activation, and health. *J. Psychosom. Res.* 60 113–124. 10.1016/j.jpsychores.2005.06.074 16439263

[B11] Bryant F. B. (2003). Savoring Beliefs Inventory (SBI): a scale fore measuring beliefs about savoring. *J. Ment. Health* 12 175–196. 10.1080/0963823031000103489

[B12] Clauss E. Hoppe A. O’Shea D. González Morales M. G. Steidle A. Michel A. (2018). Promoting personal resources and reducing exhaustion through positive work reflection among caregivers. *J. Occup. Health Psychol.* 23 127–140. 10.1037/ocp0000063 27936830

[B13] Cohen J. Cohen P. West S. G. Aiken L. S. (2003). *Applied Multiple Regression/Correlation Analysis for the Behavioral Sciences.* Mahwah, NJ: Lawrence Erlbaum.

[B14] Davis D. E. Choe E. Meyers J. Wade N. Varjas K. Gifford A. (2016). Thankful for the little things: a meta-analysis of gratitude interventions. *J. Counsel. Psychol.* 63 20–31. 10.1037/cou0000107 26575348

[B15] de Bloom J. Vaziri H. Tay L. Kujanpää M. (2020). An identity-based integrative needs model of crafting: crafting within and across life domains. *J. Appl. Psychol.* 10.1037/apl0000495 32202815

[B16] Ebert D. D. Berking M. Thiart H. Riper H. Laferton J. A. C. Cuijpers P. (2015). Restoring depleted resources: efficacy and mechanisms of change of an Internet-based unguided recovery training for better sleep and psychological detachment from work. *Health Psychol.* 34 1240–1251. 10.1037/hea0000277 26651465

[B17] Eby L. T. Maher C. P. Butts M. M. (2010). The intersection of work and family life: the role of affect. *Annu. Rev. Psychol.* 61 599–622. 10.1146/annurev.psych.093008.100422 19572785

[B18] Etzion D. Eden D. Lapidot Y. (1998). Relief from job stressors and burnout: reserve service as a respite. *J. Appl. Psychol.* 83 577–585. 10.1037/0021-9010.83.4.577 9729927

[B19] Feuerhahn N. Sonnentag S. Woll A. (2014). Exercise after work, psychological mediators, and affect: a day-level study. *Eur. J. Work Organ. Psychol.* 23 62–79. 10.1080/1359432X.2012.709965

[B20] Firoozabadi A. Uitdewilligen S. Zijlstra F. R. H. (2018). Solving problems or seeing troubles? A day-level study on the consequences of thinking about work on recovery and well-being, and the moderating role of self-regulation. *Eur. J. Work Organ. Psychol.* 27 629–641. 10.1080/1359432X.2018.1505720

[B21] Fredrickson B. L. (1998). What good are positive emotions? *J. Gen. Psychol.* 2 300–319. 10.1037/1089-2680.2.3.300 21850154 PMC3156001

[B22] Fritz C. Sonnentag S. (2006). Recovery, well-being, and performance-related outcomes: the role of workload and vacation experiences. *J. Appl. Psychol.* 91 936–945. 10.1037/0021-9010.91.4.936 16834516

[B23] Germeys L. De Gieter S. (2017). Psychological detachment mediating the daily relationship between workload and marital satisfaction. *Front. Psychol.* 7:2036. 10.3389/fpsyg.2016.02036 28101076 PMC5209365

[B24] Grant A. M. (2008). The significance of task significance: job performance effects, relational mechanisms, and boundary conditions. *J. Appl. Psychol.* 93 108–124. 10.1037/0021-9010.93.1.108 18211139

[B25] Hahn V. Binnewies C. Haun S. (2012). The role of partners for employees’ recovery during the weekend. *J. Vocat. Behav.* 80 288–298. 10.1016/j.jvb.2011.12.004

[B26] Hahn V. C. Binnewies C. Sonnentag S. Mojza E. J. (2011). Learning how to recover from job stress: effects of a recovery training program on recovery, recovery-related self-efficacy and well-being. *J. Occup. Health Psychol.* 16 202–216. 10.1037/a0022169 21463049

[B27] Hurley D. B. Kwon P. (2012). Results of a study to increase savoring the moment: differential impact on positive and negative outcomes. *J. Happiness Stud.* 13 579–588. 10.1007/s10902-011-9280-8

[B28] Ilies R. Dimotakis N. De Pater I. E. (2010). Psychological and physiological reactions to high workloads: implications for well-being. *Pers. Psychol.* 63 407–436. 10.1111/j.1744-6570.2010.01175.x

[B29] Ilies R. Keeney J. Scott B. A. (2011). Work-family interpersonal capitalization: sharing positive events at home. *Organ. Behav. Hum. Decis. Process.* 114 115–126. 10.1016/j.obhdp.2010.10.008

[B30] Ilies R. Schwind K. M. Wagner D. T. Johnson M. D. DeRue D. S. Ilgen D. R. (2007). When can employees have a family life? The effects of daily workload and affect on work-family conflict and social behavior at work. *J. Appl. Psychol.* 92 1368–1379. 10.1037/0021-9010.92.5.1368 17845091

[B31] Isen A. M. Shalker T. E. Clark M. S. Karp L. (1978). Affect, accessability of material in memory, and behavior: a cognitive loop? *J. Personal. Soc. Psychol.* 36 1–12. 10.1037/0022-3514.36.1.1 621625

[B32] Jalonen N. Kinnunen M.-L. Pulkkinen L. Kokko K. (2015). Job skill discretion and emotion control strategies as antecedents of recovery from work. *Eur. J. Work Organ. Psychol.* 24 389–401. 10.1080/1359432X.2014.914923

[B33] Judge T. A. Ilies R. (2004). Affect and job satisfaction: a study of their relationship at work and at home. *J. Appl. Psychol.* 89 661–673. 10.1037/0021-9010.89.4.661 15327352

[B34] Kempen R. Roewekaemper J. Hattrup K. Mueller K. (2019). Daily affective events and mood as antecedents of life domain conflict and enrichment: a weekly diary study. *Int. J. Stress Manag.* 26 107–119. 10.1037/str0000104

[B35] Laird J. D. Wagener J. J. Halal M. Szegda M. (1982). Remembering what you feel: effects of emotion on memory. *J. Pers. Soc. Psychol.* 42 646–657. 10.1037/0022-3514.42.4.646

[B36] Lazarus R. S. (1991). Cognition and motivation in emotion. *Am. Psychol.* 46 352–367. 10.1037/0003-066X.46.4.352 2048794

[B37] Lim S. Ilies R. Koopman J. Christoforou P. Arvey R. D. (2018). Emotional mechanisms linking incivility at work to aggression and withdrawal at home: an experience-sampling study. *J. Manag.* 44 2888–2908. 10.1177/0149206316654544

[B38] Mayer J. D. Gaschke Y. N. Braverman D. L. Evans T. W. (1992). Mood-congruent judgement is a general effect. *J. Pers. Soc. Psychol.* 63 119–132. 10.1037/0022-3514.63.1.119

[B39] Meier L. L. Cho E. (2018). Work stressors and partner social undermining: comparing negative affect and psychological detachment as mechanisms. *J. Occup. Health Psychol.* 24 359–372. 10.1037/ocp0000120 29756787

[B40] Meier L. L. Cho E. Dumani S. (2016). The effects of positive work reflection during leisure time on affective well-being: results from three diary studies. *J. Organ. Behav.* 37 255–278. 10.1002/job.2039

[B41] Michel A. Bosch C. Rexroth M. (2014). Mindfulness as a cognitive-emotional segmentation strategy: an intervention promoting work-life balance. *J. Occup. Organ. Psychol.* 87 733–754. 10.1111/joop.12072

[B42] Miron-Shatz T. Stone A. Kahneman D. (2009). Memories of yesterday’s emotions: does the valence of experience affect the memory-experience gap? *Emotion* 9 885–891. 10.1037/a0017823 20001131

[B43] Morris S. B. DeShon R. P. (2002). Combining effect size estimates in meta-analysis with repeated measures and independent-groups designs. *Psychol. Methods* 7 105–125. 10.1037//1082-989X.7.1.105 11928886

[B44] Morris W. N. (1989). *Mood: The Frame of Mind*. New York: Springer.

[B45] Neal D. T. Wood W. Labrecque J. S. Lally P. (2012). How do habits guide behavior? Perceived and actual triggers of habits in daily life. *J. Exp. Soc. Psychol.* 48 492–498. 10.1016/j.jesp.2011.10.011

[B46] Ohly S. Schmitt A. (2015). What makes us enthusiastic, angry, feeling at rest or worried? Development and validation of an affective work events taxonomy using concept mapping methodology. *J. Bus. Psychol.* 30 15–35. 10.1007/s10869-013-9328-3

[B47] Park Y. A. Fritz C. Jex S. M. (2011). Relationships between work-home segmentation and psychological detachment from work: the role of communication technology use at home. *J. Occup. Health Psychol.* 16 457–467. 10.1037/a0023594 21728434

[B48] Pindek S. Arvan M. L. Spector P. E. (2019). The stressor–strain relationship in diary studies: a meta-analysis of the within and between levels. *Work Stress* 33 1–21. 10.1080/02678373.2018.1445672

[B49] Preacher K. J. Curran P. J. Bauer D. J. (2006). Computational tools for probing interactions in multiple linear regression, multilevel modeling, and latent curve analysis. *J. Educ. Behav. Stat.* 31 437–448. 10.3102/10769986031004437

[B50] Quoidbach J. Berry E. V. Hansenne M. Mikolajczak M. (2010). Positive emotion regulation and well-being: comparing the impact of eight savoring and dampening strategies. *Pers. Individ. Differ.* 49 368–373. 10.1016/j.paid.2010.03.048

[B51] Ray R. D. Wilhelm F. H. Gross J. J. (2008). All in the mind’s eye? Anger rumination and reappraisal. *J. Pers. Soc. Psychol.* 94 133–145. 10.1037/0022-3514.94.1.133 18179323

[B52] Rodell J. B. Judge T. A. (2009). Can “good” stressors spark “bad” behaviors? The mediating role of emotions in links of challenge and hindrance stressors with citizenship and counterproductive behaviors. *J. Appl. Psychol.* 94 1438–1451. 10.1037/a0016752 19916654

[B53] Rodríguez-Muñoz A. Sanz-Vergel A. I. Antino M. Demerouti E. Bakker A. B. (2018). Positive experiences at work and daily recovery: effects on couple’s well-being. *J. Happiness Stud.* 19 1395–1413. 10.1007/s10902-017-9880-z

[B54] Rothbard N. P. Wilk S. L. (2011). Waking up on the right or wrong side of the bed: start-of-workday mood, work events, employee affect, and performance. *Acad. Manag. J.* 54 959–980. 10.5465/amj.2007.0056

[B55] Rozin P. Royzman E. B. (2001). Negativity bias, negativity dominance, and contagion. *Pers. Soc. Psychol. Rev.* 5 296–320. 10.1207/S15327957PSPR0504_2

[B56] Scherer K. R. (1999). “Appraisal theory,” in *Handbook of Cognition and Emotion*, eds Dalgleish T. Power M. J. (Chichester: Wiley), 637–663.

[B57] Semmer N. (1984). *Streßbezogene Tätigkeitsanalyse [Stress-oriented task-analysis].* Weinheim: Beltz.

[B58] Sianoja M. Kinnunen U. Mäkikangas A. Tolvanen A. (2018). Testing the direct and moderator effects of the stressor–detachment model over one year: a latent change perspective. *Work Stress* 32 357–378. 10.1080/02678373.2018.1437232

[B59] Sonnentag S. Bayer U.-V. (2005). Switching off mentally: predictors and consequences of psychological detachment from work during off-job time. *J. Occup. Health Psychol.* 10 393–414. 10.1037/1076-8998.10.4.393 16248688

[B60] Sonnentag S. Binnewies C. Mojza E. J. (2008). “Did you have a nice evening?” A day-level study on recovery experiences, sleep, and affect. *J. Appl. Psychol.* 93 674–684. 10.1037/0021-9010.93.3.674 18457495

[B61] Sonnentag S. Fritz C. (2007). The recovery experience questionnaire: development and validation of a measure assessing recuperation and unwinding from work. *J. Occup. Health Psychol.* 12 204–221. 10.1037/1076-8998.12.3.204 17638488

[B62] Sonnentag S. Fritz C. (2015). Recovery from job stress: the stressor-detachment model as an integrative framework. *J. Organ. Behav.* 36 S72–S103. 10.1002/job.1924

[B63] Sonnentag S. Grant A. M. (2012). Doing good at work feels good at home, but not right away: when and why perceived prosocial impact predicts positive affect. *Pers. Psychol.* 65 495–530. 10.1111/j.1744-6570.2012.01251.x

[B64] Sonnentag S. Starzyk A. (2015). Perceived prosocial impact, perceived situational constraints, and proactive work behavior: looking at two distinct affective pathways. *J. Organ. Behav.* 36 806–824. 10.1002/job.2005

[B65] ten Brummelhuis L. L. Bakker A. B. (2012). Staying engaged during the week: the effect of off-job activities on next day work engagement. *J. Occup. Health Psychol.* 17 445–455. 10.1037/a0029213 22799771

[B66] Volmer J. Binnewies C. Sonnentag S. Niessen C. (2012). Do social conflicts with customers at work encroach upon our private lives? A diary study. *J. Occup. Health Psychol.* 17 304–315. 10.1037/a0028454 22746368

[B67] Wang M. Liu S. Liao H. Gong Y. Kammeyer-Mueller J. Shi J. (2013). Can’t get it out of my mind: employee rumination after customer mistreatment and negative mood in the next morning. *J. Appl. Psychol.* 98 989–1004. 10.1037/a0033656 23895040

[B68] Watkins E. R. (2008). Constructive and unconstructive repetitive thought. *Psychol. Bull.* 134 163–206. 10.1037/0033-2909.134.2.163 18298268 PMC2672052

[B69] Watson D. (1988). Intraindividual and interindividual analyses of positive and negative affect: their relation to health complaints, perceived stress, and daily activities. *J. Pers. Soc. Psychol.* 54 1020–1030. 10.1037/0022-3514.54.6.1020 3397861

[B70] Watson D. Clark L. A. Tellegen A. (1988). Development and validation of brief measures of positive and negative affect: the PANAS-scales. *J. Pers. Soc. Psychol.* 54 1063–1070. 10.1037/0022-3514.54.6.1063 3397865

[B71] Weiss H. M. Cropanzano R. (1996). “Affective events theory: a theoretical discussion of the structure, causes and consequences of affective experiences at work,” in *Research in Organizational Behavior*, Vol. 18 eds Staw B. M. Cummings L. L. (Stamford, CT: JAI Press), 1–74. 10.4324/9781135048198-18

[B72] Wendsche J. Lohmann-Haislah A. (2017). A meta-analysis on antecedents and outcomes of detachment from work. *Front. Psychol.* 7:2072. 10.3389/fpsyg.2016.02072 28133454 PMC5233687

[B73] Wood W. Rünger D. (2016). Psychology of habit. *Annu. Rev. Psychol.* 67 289–314. 10.1146/annurev-psych-122414-033417 26361052

[B74] Zapf D. (1993). Stress-oriented analysis of computerized office work. *Eur. Work Organ. Psychol.* 3 85–100. 10.1080/09602009308408580

[B75] Zijlstra F. R. H. Cropley M. Rydstedt L. W. (2014). From recovery to regulation: an attempt to reconceptualize “recovery from work”. *Stress Health* 30 244–252. 10.1002/smi.2604 25100275

[B76] Zohar D. Tzischinski O. Epstein R. (2003). Effects of energy availability on immediate and delayed emotional reactions to work events. *J. Appl. Psychol.* 88 1082–1093. 10.1037/0021-9010.88.6.1082 14640818

