# Supplementary material for: To Detach or Not to Detach? Two Experimental Studies on the Affective Consequences of Detaching From Work During Non-work Time
Source: Front Psychol. 2020 Oct 16;11:560156. doi: 10.3389/fpsyg.2020.560156 (PMC7596587; doi:10.3389/fpsyg.2020.560156)
Supplement: Supplementary file 1 [file Table_1.DOCX]

Supplementary Table 1

*Manipulations in the Five Experimental Conditions*

| Hobby Condition  *[have note paper for participant ready]*  I would like to ask you to think about a hobby that you enjoy pursuing. If you do not have a specific hobby, you may think about another leisure activity you enjoy doing. Please remember a situation when you were engaged in your hobby or the leisure activity. Please remember what you have done or thought in this situation. Please take enough time to mentally focus on these situations. Use the next two minutes exclusively for exactly imagining the situations.  *[provide two minutes]*  Please note on this paper the situation and the feelings you had.  *[provide three minutes; then put paper away]*  Please continue to think about your hobby or leisure activity. Now think about the possible consequences engaging in this hobby or leisure activity can have.  *[provide two minutes]*  Please note on this paper the possible consequences engaging in this hobby or leisure activity could have.  *[provide three minutes; then put paper away]* |
| --- |
| Explicit Detachment Condition  *[have journals for participant ready]*  I would like to ask you to detach from your day at the university [Study 1] (at your job [Study 2]). Please use the following minutes to think about something different. You may – if you like – just daydream a little, but without thinking about your day at the university [Study 1] (at your job [Study 2]). Here we have some journals. You may want to read or browse through them in order to take your mind off.  *[provide journals]*  *[prevent from reading material related study or job]*  *[provide ten minutes]* |
| Negative Thinking Condition  *[have note paper for participant ready]*  I would like to ask you to think about your day at the university [Study 1] (at your job [Study 2]). Please call back to mind today’s day at the university [Study 1] (at your job [Study 2]). Please remember in particular what did not go well, and what has stressed, upset or worried you at the university [Study 1] (at your job [Study 2]) today.  In case no situation from today comes to your mind, please think about what did not go well during the past week and about events at the university [Study 1] (at work [Study 2]) that what have stressed, upset or worried you during the past week.  Please take enough time to mentally focus on these situations. Use the next two minutes exclusively for imagining the situations in detail.  *[provide two minutes]*  Please note on this paper the situation and the feelings you had.  *[provide three minutes; then put paper away]*  Please continue to think about today’s day at the university [Study 1] (at work [Study 2]) and about what has stressed, upset or worried you. Now think about the negative consequences these events or experiences can have.  *[provide two minutes]*  Please note on this paper the possible consequences today’s events or experiences could have.  *[provide three minutes; then put paper away]* |
| Positive Thinking Condition  *[have note paper for participant ready]*  I would like to ask you to think about your day at the university [Study 1] (at your job [Study 2]). Please call back to mind today’s day at the university [Study 1] (at your job [Study 2]). Please remember in particular what did go well, what has pleased or relieved you or anything else that has put you in a positive mood at the university [Study 1] (at work [Study 2]) today.”  In case no situation from today comes to your mind, please think about what went well during the past week and about events at the university [Study 1] (at work [Study 2]) that have pleased or relieved you or anything else that has put you in a positive mood during the past week.  Please take enough time to mentally focus on these situations. Use the next two minutes exclusively for imagining the situations in detail.  *[provide two minutes]*  Please note on this paper the situation and the feelings you had.  *[provide three minutes; then put paper away]*  Please continue to think about today’s day at the university [Study 1] (at work [Study 2]) and about what has pleased or relieved you or anything else that has put you in a positive mood. Now think about the positive consequences these events or experiences can have.  *[provide two minutes]*  Please note on this paper the possible consequences today’s events or experiences could have.  *[provide three minutes; then put paper away]* |
| Unspecific Thinking Condition  *[have note paper for participant ready]*  I would like to ask you to think about your day at the university [Study 1] (at your job [Study 2]). Please call back to mind today’s day at the university [Study 1] (at your job [Study 2]). Please remember in particular how your day at the university [Study 1] (at work [Study 2]) proceeded, what you have done or thought at the university [Study 1] (at work [Study 2]) today.  Please take enough time to mentally focus on these situations. Use the next two minutes exclusively for imagining the situations in detail.  *[provide two minutes]*  Please note on this paper the situation and the feelings you had.  *[provide three minutes; then put paper away]*  Please continue to think about today’s day at the university [Study 1] (at work [Study 2]) and what you have done or thought. Now think about the consequences these events or experiences can have.  *[provide two minutes]*  Please note on this paper the possible consequences today’s events or experiences could have.  *[provide three minutes; then put paper away]* |

Supplementary Table 2

*Manipulation Checks: Detailed Results (Study 1)*

| Condition | Comparison condition | Manipulation-check measure and result (Tukey’s HSD and standard error) |
| --- | --- | --- |
| Explicit detachment instruction | Hobby | Negative thinking: No significant difference (HSD = 0.083, *SE* = 0.235, *ns*)  Positive thinking: No significant difference (HSD = -0.239, *SE* = 0.258, *ns*)  Detachment experience: No significant difference (HSD = -0.429, *SE* = 0.200, *ns*)  Thinking about hobby: Lower in explicit-detachment condition (HSD = -1.590, *SE* = 0.253, *p* < .001) |
|  | Negative thinking | Negative thinking: Lower in explicit-detachment condition (HSD = -2.792, *SE* = 0.235, *p* < .001)  Positive thinking: No significant difference (HSD = -0.045, *SE* = 0.258, *ns*)  Detachment experience: Higher in explicit-detachment condition (HSD = 2.200, *SE* = 0.200, *p* < .001)  Thinking about hobby: Higher in explicit-detachment condition (HSD = 1.430, *SE* = 0.235, *p* < .001) |
|  | Positive thinking | Negative thinking: Lower in explicit-detachment condition (HSD = -0.747, *SE* = 0.233, *p* < .05)  Positive thinking: Lower in explicit-detachment condition (HSD = -2.080, *SE* = 0.255, *p* < .001)  Detachment experience: Higher in explicit-detachment condition (HSD = 1.940, *SE* = 0.200, *p* < .001)  Thinking about hobby: Higher in explicit-detachment condition (HSD = 1.300, *SE* = 0.251, *p* < .001) |
|  | Unspecific thinking | Negative thinking: Lower in explicit-detachment condition (HSD = -1.736, *SE* = 0.235, *p* < .001)  Positive thinking: Lower in explicit-detachment condition (HSD = -1.031, *SE* = 0.258, *p* < .01)  Detachment experience: Higher in explicit-detachment condition (HSD = 1.852, *SE* = 0.200, *p* < .001)  Thinking about hobby: Higher in explicit-detachment condition (HSD = 1.388, *SE* = 0.253, *p* < .001) |
| Hobby | Explicit detachment instruction | Negative thinking: No significant difference (HSD = -0.083, *SE* = 0.235, *ns*)  Positive thinking: No significant difference (HSD = 0.239, *SE* = 0.258, *ns*)  Detachment experience: No significant difference (HSD = 0.429, *SE* = 0.200, *ns*)  Thinking about hobby: Higher in hobby condition (HSD = 1.591, *SE* = 0.253, *p* < .001) |
|  | Negative thinking | Negative thinking: Lower in hobby condition (HSD = -2.875, *SE* = 0.237, *p* < .001)  Positive thinking: No significant difference (HSD = 0.194, *SE* = 0.260, *ns*)  Detachment experience: Higher in hobby condition (HSD = 2.625, *SE* = 0.200, *p* < .001)  Thinking about hobby: Higher in hobby condition (HSD = 3.021, *SE* = 0.256, *p* < .001) |
|  | Positive thinking | Negative thinking: Lower in hobby condition (HSD = -0.830, *SE* = 0.235, *p* < .01)  Positive thinking: Lower in hobby condition (HSD = -1.841, *SE* = 0.258, *p* < .001)  Detachment experience: Higher in hobby condition (HSD = 1.369, *SE* = 0.200, *p* < .001)  Thinking about hobby: Higher in hobby condition (HSD = 2.891, *SE* = 0.253, *p* < .001) |
|  | Unspecific thinking | Negative thinking: Lower in hobby condition (HSD = -1.819, *SE* = 0.235, *p* < .001)  Positive thinking: Lower in hobby condition (HSD = -0.792, *SE* = 0.260, *p* < .05)  Detachment experience: Higher in hobby condition (HSD = 2.281, *SE* = 0.200, *p* < .001)  Thinking about hobby: Higher in hobby condition (HSD = 1.979, *SE* = 0.256, *p* < .001) |
| Negative thinking | Explicit detachment instruction | Negative thinking: Higher in negative-thinking condition (HSD = 2.792, *SE* = 0.235, *p* < .001)  Positive thinking: No significant difference (HSD = 0.045, *SE* = 0.258, *ns*)  Detachment experience: Lower in negative-thinking condition (HSD = -2.200, *SE* = 0.200, *p* < .001)  Thinking about hobby: Lower in negative-thinking condition (HSD = -1.430, *SE* = 0.253, *p* < .001) |
|  | Hobby | Negative thinking: Higher in negative-thinking condition (HSD = 2.875, *SE* = 0.237, *p* < .001)  Positive thinking: No significant difference (HSD = -0.194, *SE* = 0.260, *ns*)  Detachment experience: Lower in negative-thinking condition (HSD = -2.625, *SE* = 0.200, *p* < .001)  Thinking about hobby: Lower in negative-thinking condition (HSD = -3.031, *SE* = 0.256, *p* < .001) |
|  | Positive thinking | Negative thinking: Higher in negative-thinking condition (HSD = 2.045, *SE* = 0.235, *p* < .001)  Positive thinking: Lower in negative-thinking condition (HSD = -2.035, *SE* = 0.258, *p* < .001)  Detachment experience: No significant difference (HSD = -0.256, *SE* = 0.200, *ns*)  Thinking about hobby: No significant difference (HSD = -0.130, *SE* = 0.253, *ns*) |
|  | Unspecific thinking | Negative thinking: Higher in negative-thinking condition (HSD = 1.056, *SE* = 0.237, *p* < .001)  Positive thinking: Lower in negative-thinking condition (HSD = -0.986, *SE* = 0.260, *p* < .01)  Detachment experience: No significant difference (HSD = -0.344, *SE* = 0.200, *ns*)  Thinking about hobby: No significant difference (HSD = -0.042, *SE* = 0.256, *ns*) |
| Positive thinking | Explicit detachment instruction | Negative thinking: Higher in positive-thinking condition (HSD = 0.747, *SE* = 0.233, *p* < .05)  Positive thinking: Higher in positive-thinking condition (HSD = 2.080, *SE* = 0.255, *p* < .001)  Detachment experience: Lower in positive-thinking condition (HSD = -1.940 *SE* = 0.200, *p* < .001)  Thinking about hobby: Lower in positive-thinking condition (HSD = -1.300, *SE* = 0.251, *p* < .001) |
|  | Hobby | Negative thinking: Higher in positive-thinking condition (HSD = 0.830, *SE* = 0.235, *p* < .01))  Positive thinking: Higher in positive-thinking condition (HSD = 1.841, *SE* = 0.258, *p* < .001)  Detachment experience: Lower in positive-thinking condition (HSD = -2.369, *SE* = 0.200, *p* < .001)  Thinking about hobby: Lower in positive-thinking condition (HSD = -2.891, *SE* = 0.253, *p* < .001) |
|  | Negative thinking | Negative thinking: Lower in positive-thinking condition (HSD = -2.045, *SE* = 0.235, *p* < .001)  Positive thinking: Higher in positive-thinking condition (HSD = 2.035, *SE* = 0.258, *p* < .001)  Detachment experience: No significant difference (HSD = 0.256, *SE* = 0.200, *ns*)  Thinking about hobby: No significant difference (HSD = 0.130, *SE* = 0.253, *ns*) |
|  | Unspecific thinking | Negative thinking: Lower in positive-thinking condition (HSD = -0.989, *SE* = 0.235, *p* < .001)  Positive thinking: Higher in positive-thinking condition (HSD = 1.049, *SE* = 0.258, *p* < .01)  Detachment experience: No significant difference (HSD = -0.088, *SE* = 0.200, *ns*)  Thinking about hobby: No significant difference (HSD = 0.088, *SE* = 0.253, *ns*) |
| Unspecific thinking | Explicit detachment instruction | Negative thinking: Higher in unspecific-thinking condition (HSD = 1.736, *SE* = 0.235, *p* < .001)  Positive thinking: Higher in unspecific-thinking condition (HSD = 1.031, *SE* = 0.258, *p* < .01)  Detachment experience: Lower in unspecific-thinking condition (HSD = -1.852, *SE* = 0.200, *p* < .001)  Thinking about hobby: Lower in unspecific-thinking condition (HSD = -1.388, *SE* = 0.253, *p* < .001) |
|  | Hobby | Negative thinking: Higher in unspecific-thinking condition (HSD = 1.819, *SE* = 0.237, *p* < .001)  Positive thinking: Higher in unspecific-thinking condition (HSD = 0.792, *SE* = 0.260, *p* < .05)  Detachment experience: Lower in unspecific-thinking condition (HSD = -2.281, *SE* = 0.200, *p* < .001)  Thinking about hobby: Lower in unspecific-thinking condition (HSD = -2.979, *SE* = 0.256, *p* < .001) |
|  | Negative thinking | Negative thinking: Lower in unspecific-thinking condition (HSD = -1.056, *SE* = 0.237, *p* < .001)  Positive thinking: Higher in unspecific-thinking condition (HSD = 0986, *SE* = 0.260, *p* < .01)  Detachment experience: No significant difference (HSD = 0.344, *SE* = 0.200, *ns*)  Thinking about hobby: No significant difference (HSD = 0.042, *SE* = 0.256, *ns*) |
|  | Positive thinking | Negative thinking: Higher in unspecific-thinking condition (HSD = 0.989, *SE* = 0.235, *p* < .001)  Positive thinking: Lower in unspecific-thinking condition (HSD = -1.049, *SE* = 0.258, *p* < .01)  Detachment experience: No significant difference (HSD = 0.088, *SE* = 0.200, *ns*)  Thinking about hobby: No significant difference (HSD = -0.088, *SE* = 0.253, *ns*) |

Supplementary Table 3

*Manipulation Checks: Detailed Results (Study 2)*

|  | Negative affect | | Positive affect |
| --- | --- | --- | --- |
| Condition | Comparison condition | Manipulation-check measure and result (Tukey’s HSD and standard error) | |
| Explicit detachment instruction | Hobby | Negative thinking: No significant difference (HSD = -0.688, *SE* = 0.251, *ns*)  Positive thinking: Lower in explicit-detachment condition (HSD = -1.036, *SE* = 0.247, *p* < .001)  Detachment experience: No significant difference (HSD = 0.408, *SE* = 0.224, *ns*)  Thinking about hobby: Lower in explicit-detachment condition (HSD = -1.549, *SE* = 0.222, *p* < .001) | |
|  | Negative thinking | Negative thinking: Lower in explicit-detachment condition (HSD = -2.646, *SE* = 0.252, *p* < .001)  Positive thinking: Lower in explicit-detachment condition (HSD = -0.708, *SE* = 0.249, *p* < .05)  Detachment experience: Higher in explicit-detachment condition (HSD = 2.055, *SE* = 0.226, *p* < .001)  Thinking about hobby: Higher in explicit-detachment condition (HSD = 1.625, *SE* = 0.224, *p* < .001) | |
|  | Positive thinking | Negative thinking: Lower in explicit-detachment condition (HSD = -0.816, *SE* = 0.255, *p* < .05)  Positive thinking: Lower in explicit-detachment condition (HSD = -2.682, *SE* = 0.251, *p* < .001)  Detachment experience: Higher in explicit-detachment condition (HSD = 2.248, *SE* = 0.228, *p* < .001)  Thinking about hobby: Higher in explicit-detachment condition (HSD = 1.487, *SE* = 0.225, *p* < .001) | |
|  | Unspecific thinking | Negative thinking: Lower in explicit-detachment condition (HSD = -1.493, *SE* = 0.255, *p* < .001)  Positive thinking: Lower in explicit-detachment condition (HSD = -1.736, *SE* = 0.251, *p* < .001)  Detachment experience: Higher in explicit-detachment condition (HSD = 1.974, *SE* = 0.228, *p* < .001)  Thinking about hobby: Higher in explicit-detachment condition (HSD = 1.487, *SE* = 0.225, *p* < .001) | |
| Hobby | Explicit detachment instruction | Negative thinking: No significant difference (HSD = 0.688, *SE* = 0.251, *ns*)  Positive thinking: Higher in hobby condition (HSD = 1.036, *SE* = 0.247, *p* < .001)  Detachment experience: No significant difference (HSD = -0.408, *SE* = 0.224, *ns*)  Thinking about hobby: Higher in hobby condition (HSD = 1.549, *SE* = 0.222, *p* < .001) | |
|  | Negative thinking | Negative thinking: Lower in hobby condition (HSD = -1.957, *SE* = 0.251, *p* < .001)  Positive thinking: No significant difference (HSD = 0.328, *SE* = 0.247, *ns*)  Detachment experience: Higher in hobby condition (HSD = 1.647, *SE* = 0.224, *p* < .001)  Thinking about hobby: Higher in hobby condition (HSD = 3.174, *SE* = 0.222, *p* < .001) | |
|  | Positive thinking | Negative thinking: No significant difference (HSD = -0.127, *SE* = 0.253, *ns*)  Positive thinking: Lower in hobby condition (HSD = -1.646, *SE* = 0.249, *p* < .001)  Detachment experience: Higher in hobby condition (HSD = 1.841, *SE* = 0.226, *p* < .001)  Thinking about hobby: Higher in hobby condition (HSD = 3.036, *SE* = 0.224, *p* < .001) | |
|  | Unspecific thinking | Negative thinking: Lower in hobby condition (HSD = -0.805, *SE* = 0.253, *p* < .05)  Positive thinking: Lower in hobby condition (HSD = -0.700, *SE* = 0.249, *p* < .05)  Detachment experience: Higher in hobby condition (HSD = 1.566, *SE* = 0.226, *p* < .001)  Thinking about hobby: Higher in hobby condition (HSD = 3.036, *SE* = 0.224, *p* < .001) | |
| Negative thinking | Explicit detachment instruction | Negative thinking: Higher in negative-thinking condition (HSD = 2.646, *SE* = 0.252, *p* < .001)  Positive thinking: Higher in negative-thinking condition (HSD = 0.708, *SE* = 0.249, *p* < .05)  Detachment experience: Lower in negative-thinking condition (HSD = -2.055, *SE* = 0.226, *p* < .001)  Thinking about hobby: Lower in negative-thinking condition (HSD = -1.625, *SE* = 0.224, *p* < .001) | |
|  | Hobby | Negative thinking: Higher in negative-thinking condition (HSD = 1.957, *SE* = 0.251, *p* < .001)  Positive thinking: No significant difference (HSD = -0.328, *SE* = 0.247, *ns*)  Detachment experience: Lower in negative-thinking condition (HSD = -1.647, *SE* = 0.224, *p* < .001)  Thinking about hobby: Lower in negative-thinking condition (HSD = -3.174, *SE* = 0.222, *p* < .001) | |
|  | Positive thinking | Negative thinking: Higher in negative-thinking condition (HSD = 1.830, *SE* = 0.255, *p* < .001)  Positive thinking: Lower in negative-thinking condition (HSD = -1.974, *SE* = 0.251, *p* < .001)  Detachment experience: No significant difference (HSD = 0.194, *SE* = 0.228, *ns*)  Thinking about hobby: No significant difference (HSD = -0.138, *SE* = 0.225, *ns*) | |
|  | Unspecific thinking | Negative thinking: Higher in negative-thinking condition (HSD = 1.153, *SE* = 0.255, *p* < .001)  Positive thinking: Lower in negative-thinking condition (HSD = -1.028, *SE* = 0.251, *p* < .01)  Detachment experience: No significant difference (HSD = -0.080, *SE* = 0.228, *ns*)  Thinking about hobby: No significant difference (HSD = -0.138, *SE* = 0.225, *ns*) | |
| Positive thinking | Explicit detachment instruction | Negative thinking: Higher in positive-thinking condition (HSD = 0.816, *SE* = 0.255, *p* < .05)  Positive thinking: Higher in positive-thinking condition (HSD = 2.682, *SE* = 0.251, *p* < .001)  Detachment experience: Lower in positive-thinking condition (HSD = -2.248, *SE* = 0.228, *p* < .001)  Thinking about hobby: Lower in positive-thinking condition (HSD = -1.487, *SE* = 0.225, *p* < .001) | |
|  | Hobby | Negative thinking: No significant difference (HSD = 0.127, *SE* = 0.253, *ns*)  Positive thinking: Higher in positive-thinking condition (HSD = 1.646, *SE* = 0.249, *p* < .001)  Detachment experience: Lower in positive-thinking condition (HSD = -1.841, *SE* = 0.226, *p* < .001)  Thinking about hobby: Lower in positive-thinking condition (HSD = -3.036, *SE* = 0.224, *p* < .001) | |
|  | Negative thinking | Negative thinking: Lower in positive-thinking condition (HSD = -1.830, *SE* = 0.255, *p* < .001)  Positive thinking: Higher in positive-thinking condition (HSD = 1.974, *SE* = 0.251, *p* < .001)  Detachment experience: No significant difference (HSD = -0.194, *SE* = 0.228, *ns*)  Thinking about hobby: No significant difference (HSD = 0.138, *SE* = 0.225, *ns*) | |
|  | Unspecific thinking | Negative thinking: No significant difference (HSD = -0.677, *SE* = 0.257, *ns*)  Positive thinking: Higher in positive-thinking condition (HSD = 0.946, *SE* = 0.253, *p* < .01)  Detachment experience: No significant difference (HSD = -0.274, *SE* = 0.230, *ns*)  Thinking about hobby: No significant difference (HSD = 0.000, *SE* = 0.227, *ns*) | |
| Unspecific thinking | Explicit detachment instruction | Negative thinking: Higher in unspecific-thinking condition (HSD = 1.493, *SE* = 0.255, *p* < .001)  Positive thinking: Higher in unspecific-thinking condition (HSD = 1.736, *SE* = 0.251, *p* < .001)  Detachment experience: Lower in unspecific-thinking condition (HSD = -1.974, *SE* = 0.228, *p* < .001)  Thinking about hobby: Lower in unspecific-thinking condition (HSD = -1.487, *SE* = 0.225, *p* < .001) | |
|  | Hobby | Negative thinking: Higher in unspecific-thinking condition (HSD = 0.805, *SE* = 0.253, *p* < .05)  Positive thinking: Higher in unspecific-thinking condition (HSD = 0.700, *SE* = 0.249, *p* < .05)  Detachment experience: Lower in unspecific-thinking condition (HSD = -1.566, *SE* = 0.226, *p* < .001)  Thinking about hobby: Lower in unspecific-thinking condition (HSD = -3.036, *SE* = 0.224, *p* < .001) | |
|  | Negative thinking | Negative thinking: Lower in unspecific-thinking condition (HSD = -1.153, *SE* = 0.255, *p* < .001)  Positive thinking: Higher in unspecific-thinking condition (HSD = 1.028, *SE* = 0.251, *p* < .01)  Detachment experience: No significant difference (HSD = 0.080, *SE* = 0.228, *ns*)  Thinking about hobby: No significant difference (HSD = 0.138, *SE* = 0.225, *ns*) | |
|  | Positive thinking | Negative thinking: Negative thinking: No significant difference (HSD = 0.677, *SE* = 0.257, *ns*)  Positive thinking: Lower in unspecific-thinking condition (HSD = -0.946, *SE* = 0.253, *p* < .01)  Detachment experience: No significant difference (HSD = 0.274, *SE* = 0.230, *ns*)  Thinking about hobby: No significant difference (HSD = 0.000, *SE* = 0.227, *ns*) | |
